# Comprehensive functional characterization of complement factor I rare variant genotypes identified in the SCOPE geographic atrophy cohort

**DOI:** 10.1016/j.jbc.2024.107452

**Published:** 2024-06-07

**Authors:** Thomas M. Hallam, Anneliza Andreadi, Scott J. Sharp, Vicky Brocklebank, Emanuela Gardenal, Anna Dreismann, SCOPE Study Group, Rashi Arora, Marcus Dennis, Christina Flaxel, Edward Hall, Carel Hoyng, Peter Charbel Issa, Nicolas Leveziel, Fanni Molnár, Rafael Navarro, Todd Schneiderman, David Steel, Ramin Tadayoni, Tongalp Tezel, Michel Weber, Andrew J. Lotery, Kevin J. Marchbank, Claire L. Harris, Amy V. Jones, David Kavanagh

**Affiliations:** 1 Gyroscope Therapeutics Limited, A Novartis Company, London, UK; 2 Complement Therapeutics Research Group, Translational and Clinical Research Institute, Newcastle University, Newcastle upon Tyne, UK; 3 National Renal Complement Therapeutics Centre, Royal Victoria Infirmary, Newcastle upon Tyne, UK; 4 Clinical and Experimental Sciences, Faculty of Medicine, University Hospital Southampton, University of Southampton, Southampton, UK; 5 Biosciences Institute, Newcastle University, Newcastle upon Tyne, UK

**Keywords:** complement, complement system, enzyme mutation, retinal degeneration, innate immunity, complement factor I, age-related macular degeneration (AMD), atypical hemolytic uremic syndrome (aHUS), C3 glomerulopathy(C3G)

## Abstract

Rare variants (RVs) in the gene encoding the regulatory enzyme complement factor I (*CFI*; FI) that reduce protein function or levels increase age-related macular degeneration risk. A total of 3357 subjects underwent screening in the SCOPE natural history study for geographic atrophy secondary to age-related macular degeneration, including *CFI* sequencing and serum FI measurement. Eleven *CFI* RV genotypes that were challenging to categorize as type I (low serum level) or type II (normal serum level, reduced enzymatic function) were characterized in the context of pure FI protein in C3b and C4b fluid phase cleavage assays and a novel bead-based functional assay (BBFA) of C3b cleavage. Four variants predicted or previously characterized as benign were analyzed by BBFA for comparison. In all, three variants (W51S, C67R, and I370T) resulted in low expression. Furthermore, four variants (P64L, R339Q, G527V, and P528T) were identified as being highly deleterious with IC50s for C3b breakdown >1 log increased versus the WT protein, while two variants (K476E and R474Q) were ∼1 log reduced in function. Meanwhile, six variants (P50A, T203I, K441R, E548Q, P553S, and S570T) had IC50s similar to WT. Odds ratios and BBFA IC50s were positively correlated (r = 0.76, *p* < 0.01), while odds ratios versus combined annotation dependent depletion (CADD) scores were not (r = 0.43, *p* = 0.16). Overall, 15 *CFI RV*s were functionally characterized which may aid future patient stratification for complement-targeted therapies. Pure protein *in vitro* analysis remains the gold standard for determining the functional consequence of *CFI RV*s.

Age-related macular degeneration (AMD) is a common cause of irreversible blindness, with prevalence in the developed world higher than any other cause, ∼290 million individuals could be affected globally by 2040 with no signs of slowing incidence rates (1, 2). Complex pathophysiology and distinct subphenotypes suggest personalized approaches to AMD treatment may be optimal (3). Classified into “wet” and “dry” disease states dependent on angiogenic features, individuals can be affected by either or both forms in one or two eyes (4). The “wet” exudative form of the disease is currently treated by anti-vascular endothelial growth factor therapy although its success has been limited, with visual gains not maintained long-term (5, 6). Currently, there are two complement inhibitory therapeutics recently approved by the Food and Drug Administration for treatment of the dry form of the disease characterized by loss of cells in the macula area of the eye and described clinically as geographic atrophy (GA) (7, 8). However promising, these therapies have currently exhibited limited functional benefit (9). Common single nucleotide polymorphisms or rare variants (RVs) in genes of the complement system, and pathways of extracellular matrix remodeling, metabolic homeostasis, and immune cell regulation, have all been strongly associated with AMD in genome (10, 11) and transcriptome-wide association studies (12). The strongest genetic risk loci are located at chr 1 and 10, encoding complement factor H (*CFH*), and *HTRA1/ARMS2*, respectively. Strong risk loci also map to genes encoding complement components C3, C9 (13), factor B (*CFB*; FB), and factor I (*CFI*; FI). Historical studies dating back to 2005 reported protein coding and noncoding single nucleotide polymorphism and/or risk haplotypes in *CFH*, *C3*, and *CFB* associated with AMD (14, 15, 16, 17, 18, 19). Further reports have revealed the importance of the CFHRs and FHL-1 proteins also encoded at the chr1 locus alongside *CFH* (20, 21). These results collectively implicate the alternative pathway (AP) of complement activation in AMD pathophysiology.

The AP is an amplification loop centered at the component C3, with its cleavage product, C3b, forming part of the C3 cleaving enzyme with FB breakdown product Bb, the C3 convertase (C3bBb). In a constant state of surveillance for foreign or dying cell surfaces and immune debris, and with a low level of ”tick-over” activation occurring constitutively (22, 23), the AP requires tight regulation to halt runaway turnover that would drive inflammation through the anaphylatoxin C5a and immune cell antigens C3b and iC3b. This control is achieved by the expression of regulatory proteins, including FH (24, 25) and FI.

FI is the only regulatory enzyme of the AP (26, 27, 28). FI cleaves exclusively C3b (and C4b of the classical pathway (CP)) when assisted by cofactors FH (AP only), membrane cofactor protein, and complement receptor 1 (AP and CP) (24, 25, 29, 30, 31, 32). *CFI* was first associated with AMD risk in 2009 (33), followed by 2013 studies of targeted and exome-wide sequencing (34, 35). Importantly, 2015, 2020, and 2022 studies in distinct European and US cohorts revealed that rare *CFI* variants that resulted in haploinsufficiency (low antigenic levels in serum, referred to as “type I” variants) were strongly associated with advanced AMD with odd ratios (ORs) >10 (36, 37) and ORs 3.1 to 7.8 (38). Further investigation has shown that *CFI* RV with abrogated function but normal levels (referred to as “Type II” variants) are also identified in a small proportion of AMD (39); almost 50% of *CFI* RVs identified in AMD are secreted at a similar level to WT in *in vitro* analyses (40).

Further investigations of the role *CFI* RVs play in AMD have identified correlations with increased macular thinning in the UK biobank (41), and more rapid progression to advanced AMD (42). Recent studies applying mendelian randomization to triangulate *CFI* genetic advanced AMD association data and systemic FI levels revealed that a 1 SD decrease in FI level led to an approximately 50% increased odds of developing advanced AMD (43, 44). These observations led to the pursuit of FI replacement as a therapeutic strategy for patients with GA, with the *CFI* RV positive subpopulation of GA being hypothesized as maximally responsive to FI supplementation therapy. To this end, subretinal adeno-associated virus gene therapy driving expression of human FI protein to slow macular degeneration in GA was tested in PhI/II clinical trials (45). Characterizing the phenotypic consequence of *CFI* RV genotypes identified in AMD into groups of shared (dys)function is an important approach to support patient stratification efforts and helps characterize a potentially important subpopulation of GA.

In addition to isolation in AMD, *CFI* RVs are also observed in rare renal and neurological diseases such as atypical hemolytic uremic syndrome (aHUS) (46) (with mutation dependent treatment differences observed (47), C3 glomerulopathy/membranoproliferative glomerulonephritis (48), and deficiency of factor I with cerebral inflammation syndrome, in addition to complete *CFI* immunodeficiency (27). A substantial number of these RVs are currently uncharacterized. However, clinical variant annotation databases (*e.g.* OMIM, ClinVar) used to support variant pathogenicity assignment and guide clinical decision-making lack sufficient coverage for the breadth of *CFI* RV identified. To address this gap, this study undertook functional investigation of uncharacterized *CFI* RV identified in subjects with GA secondary to AMD, being screened for eligibility to participate in SCOPE, a natural history study. Subjects with *CFI* RV were tested for serum FI levels, to help classify the underlying (dys) function of the *CFI* RV genotype, as a type I, a type II, or a variant with no functional effect (benign). A total of 15 *CFI* RV genotypes identified in GA subjects underwent comprehensive *in vitro* investigation, using previously published methods (49) for generating pure proteins to avoid confounding effects of improper processing and heterozygous serum analysis in subsequent characterization with standard and novel functional assays of enzymatic activity for C3b and C4b. These data add to the growing knowledge bank on dysfunctional *CFI* RV genotypes, which may help clinicians in the diagnosis of complement-related diseases and future clinical development of complement-targeted therapies. Comparison of *in vitro* findings on *CFI* RV genotype function with paired *in silico* prediction of variant deleteriousness, indicated that *in vitro* characterization of pure protein should remain the gold standard for understanding the consequence of novel variants affecting the FI protein and thus for improving patient stratification for clinical trials.

## Results

### Identification and initial classification of *CFI* RVs in SCOPE

**Figure 1:**
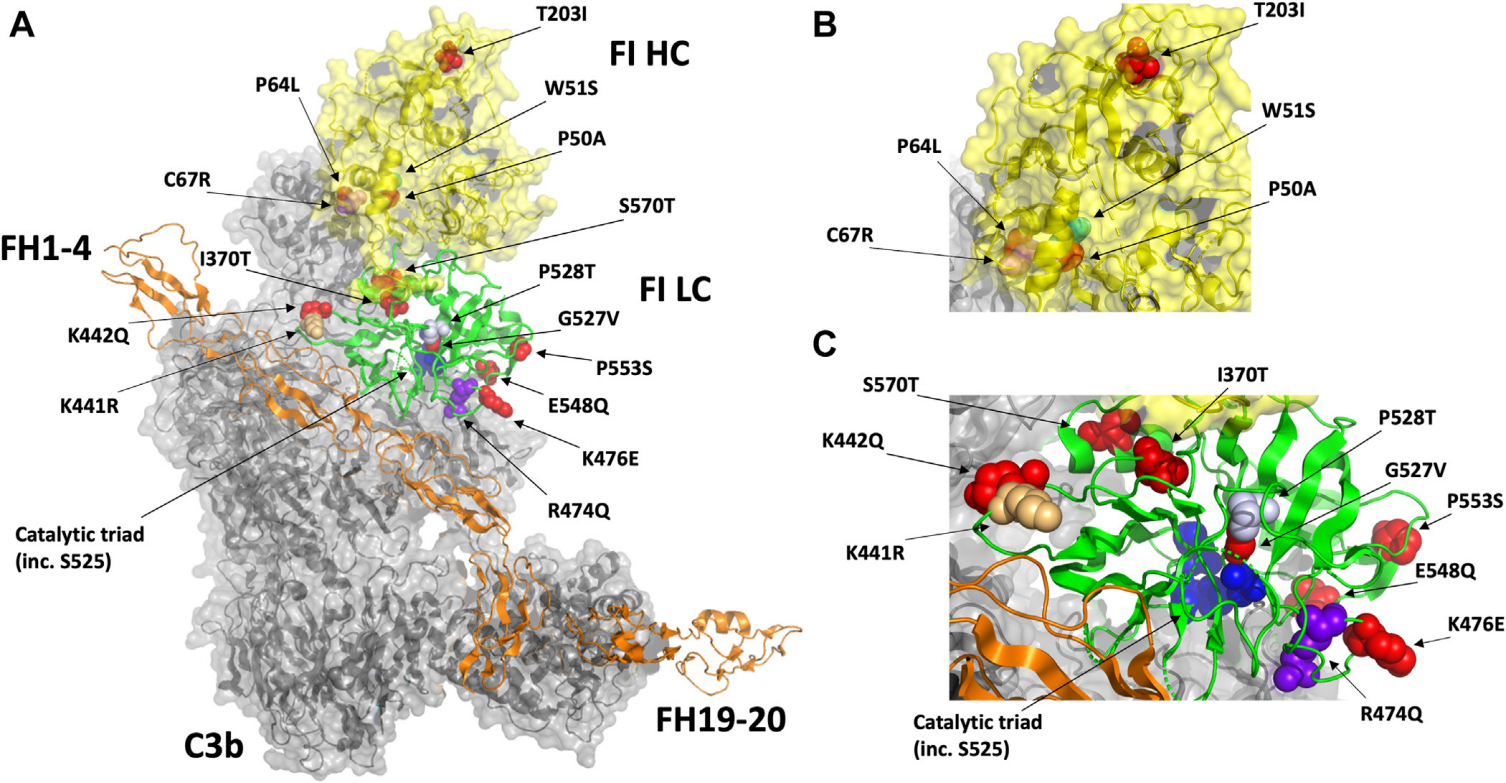
**SCOPE FI variants selected for production, position within the AP regulatorytrimolecular complex.***A*, three-dimensional structure of the AP regulatory trimolecular complex of FH1-4_19 to 20 (*orange*), C3b (*gray*), and Factor I (heavy chain (HC): *yellow*, and light chain (LC): *green*), amino acid with identified variation within the cohort (colored (*red*, *green*, *gray*, or *purple spheres*), catalytic triad (*blue spheres*). *B*, zoomed in on HC. *C*, zoomed in on LC. Figures were generated using PyMOL v2.5.4 (Schrodinger, LLC) and PDB structure 5o32 (65). AP, alternative pathway; PDB, Protein Data Bank.

Targeted sequencing of the *CFI* coding region identified that 236 out of 3357 (7.0%) GA subjects were positive for a *CFI* RV (coding change, ≤1% minor allele frequency [MAF]). A total of 71 different genotypes were identified, with three subjects positive for two heterozygous *CFI* RVs each. *CFI* RVs were significantly enriched in GA subjects being screened for SCOPE (OR 4.27-993), when comparing allele frequencies to those observed in a large collection of European individuals (non-Finnish Europeans; NFEs) from GnomAD database (n = 622,057) (50). GnomAD is a publicly available resource providing precise genetic variant allele frequency data from large body of different ethnic populations, and has been used as the control in various genetic association studies, when paired controls are unavailable, as was the case with the SCOPE study (51). Serum FI measurement was undertaken where possible for subjects carrying *CFI* RVs, where levels were compared to a predefined threshold of 15.6 μg/ml defining “low” FI (43). Initially, a total of 2.8% of subjects carried genotypes categorized as type I (n = 85/236), with the remaining 4.2% of subjects carrying genotypes associated with normal, or uncertain antigenic consequence at time of selection. From this latter group, 11 genotypes were taken forward for functional characterization alongside designed inactive S525A and benign K442Q (Table 1) based on analysis of 3D structural topology favoring proximity to the FI catalytic triad or putative binding sites within the AP regulatory trimolecular complex (TMC) of C3b:FH:FI (all but R339Q (linker variant) shown in Figure 1), substantial changes to amino acid side chain properties, allele frequency, and lack of prior published functional characterization at time of selection. The selected variants were produced as pure FI protein and underwent fluid phase cofactor assays for C3b and C4b prior to further analysis. An additional four secreted *CFI* RVs (T203I, K441R, E548Q, and P553S) that were previously characterized (K441R and P553S) (27, 49), or predicted to be of little functional consequence (T203I and E548Q), were also selected for investigation to add confidence by comparing function *in vitro* to WT and inactive S525A FI in a single bead-based functional assay (BBFA) of C3b cleavage.

**Table 1:** *CFI* RV identified in subjects with GA being screened for enrollment into SCOPE (n = 3357)

Transcript consequence	Protein change	rsID	Allele number (SCOPE)	Allele frequency (SCOPE)	Allele frequency (GnomAD NFE)	OR	95% CI	*p* value
c.1737G>C	S570T	rs200973120	3	8.937E-04	2.093E-04	4.27	1.367–13.34	0.04
c.1685C>T	P553S	rs113460688	13	0.0038725	0.0033009	1.18	0.68–2.03	0.6617
c.1670G>C	E548Q	rs7437875	1	0.00029789	0.0001873	1.591	0.2231–11.35	0.4677
c.1610C>A	P528T	No rsID	1	2.979E-04	NA	NA	NA	NA
c.1608G>T	G527V	rs1436775364	1	2.979E-04	0.000E+00	993.90	40.48–24,401	0.003
c.1601T>G	S525A	No rsID	NA	NA	NA	NA	NA	NA
c.1454A>G	K476E	No rsID	1	2.979E-04	NA	NA	NA	NA
c.1449G>A	R474Q	rs765956155	1	2.979E-04	2.797E-05	10.65	1.46–77.92	0.09
c.1352A>C	K442Q	rs774830806	NA	NA	NA	NA	NA	NA
c.1350A>G	K441R	rs41278047	37	0.0110217	0.0015059	7.40	5.34–10.27	<0.0001
c.1137T>C	pI370T	rs1167888427	1	2.979E-04	0.000E+00	312.90	12.75–7683	0.01
c.1044G>A	R339Q	rs773085612	3	8.937E-04	1.441E-05	62.06	18.18–211.9	<0.0001
c.636C>T	T203I	rs138346388	2	0.00059577	0.0002331	2.558	0.6362–10.28	0.1859
c.227T>C	C67R	rs1727799010	1	2.979E-04	NA	NA	NA	NA
c.219C>T	P64L	rs773187287	1	2.979E-04	4.068E-05	7.33	1.01–53.09	0.13
c.180G>C	W51S	No rsID	1	2.979E-04	5.250E-06	56.76	6.342–507.9	0.02
c.176C>G	P50A	rs144082872	6	1.787E-03	8.306E-05	21.56	9.447–49.19	<0.0001

Coordinates for *CFI* RV genotype according to transcript and protein are provided alongside genotype Reference SNP cluster ID (rsID). *CFI* RV allele number and frequency in SCOPE GA and GnomAD population control database of non-Finnish Europeans (NFE) are listed. Odds ratio (OR) with *p* < 0.05 were considered statistically significant. CI, confidence interval, NA, not available due to lack of genotype data in GnomAD.

SNP, single nucleotide polymorphisms.

### Expression and secretion of SCOPE *CFI* RVs *versus* WT

**Figure 2:**
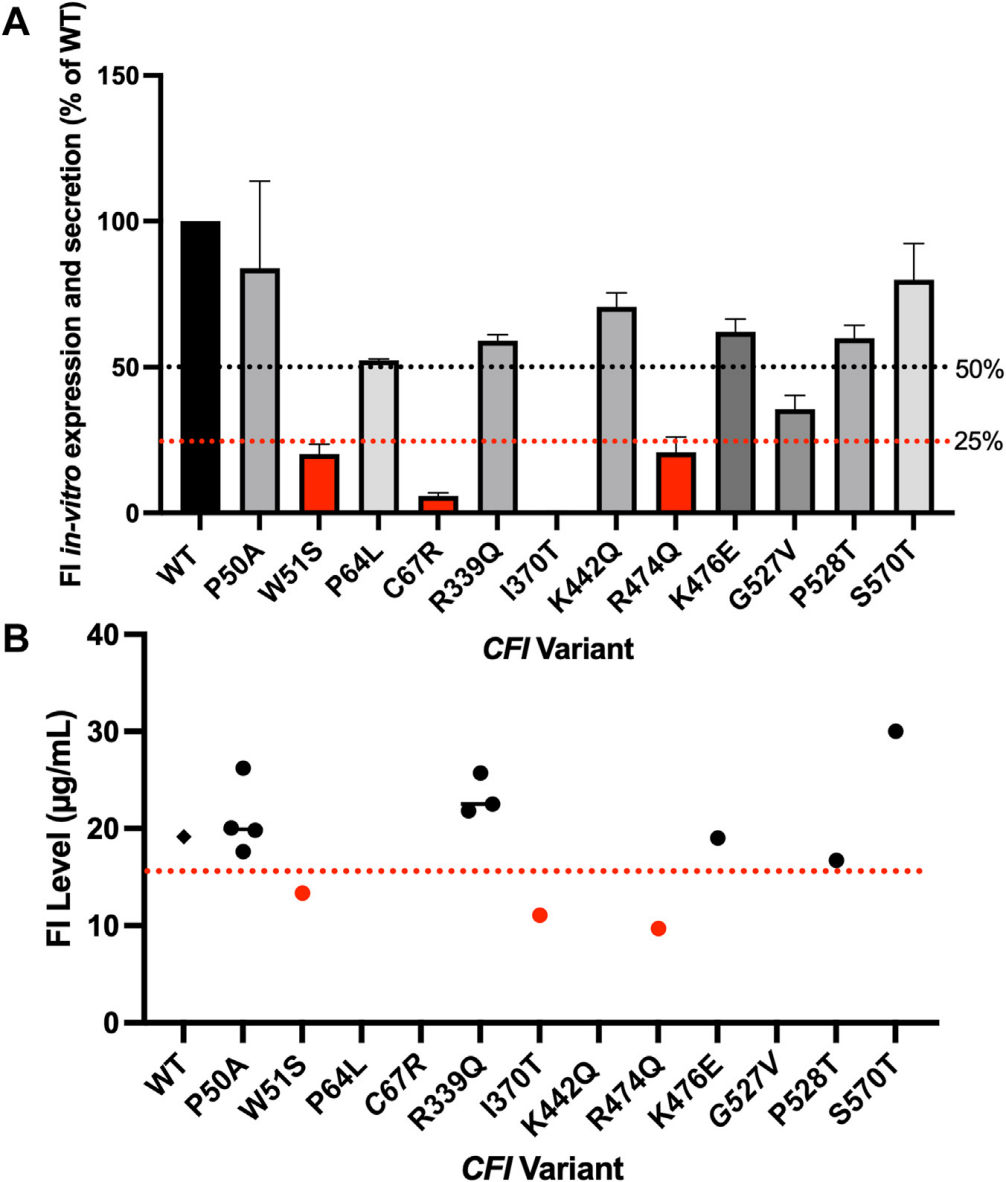
**Antigenic assessment of FI variants.***A*, recombinant expression and secretion analysis of FI variants. In six well-plates, HEK293T cells were transfected using the standard method with plasmids containing cDNA for each FI variant and incubated for 72 h in the absence of hygromycin (N = 10 for WT, minimum of N = 3 for each variant). ELISA was used to determine the concentration, shown here as the percentage of WT. The mean and standard deviation are displayed for each variant. *Lines* represent 50% and 25% of WT level, and *red bars* represent those below 25% expression *versus* WT. *B*, antigenic levels for FI Variants in serum. Plotted by *circles* are the FI serum levels (μg/ml) detected in the serum samples of individuals in the SCOPE cohort carrying the *CFI* rare variants investigated in this study. The *black diamond* for “WT” represents the mean FI level for the control cohort (19.1 μg/ml). The *red dotted line* represents the low-level cutoff point (15.6 μg/ml). No measurement was performed for the carriers of three variants (P64L, C67R and G527V) and K442Q was a designed mutant. *Red dots* represent those variants with FI levels below the low-level cutoff. cDNA, complementary DNA; CFI, complement factor I.

Established mechanisms for type I *CFI* RV dysfunction leading to reduced production of FI protein can be a consequence of inefficient translation or a failure of misfolded protein secretion by the cell into the local environment (27). The latter mechanism was investigated in the SCOPE *CFI* RVs using *in vitro* expression of recombinant FI from complementary DNA (cDNA) plasmids. To test for low expression and/or secretion, independent small-scale transient transfection tests were run, and an ELISA was used to detect protein in the supernatant for each variant-expressing plasmid normalized to expression of WT protein from the WT cDNA plasmid (minimum N = 3) (Fig. 2*A*). In this setting, it is expected that a nonsecreted variant would result in low to no FI protein in the supernatant. As such, the majority of variants that were successfully purified were expressed and secreted at >50% of the rate of the WT protein; however, G527V was ∼35% of WT. All three variants that could not be produced for functional analysis (W51S, C67R, and I370T) were expressed at <25% *versus* the WT protein, with a fourth (R474Q) variant only generated after production scale-up. Updated and concordant serum levels were obtained from subjects carrying three of the four variants that could not be made and/or exhibited low *in vitro* expression (W51S, I370T, and R474Q), low antigenic protein levels were identified in serum for one RV carrier in the cohort for each variant (Fig. 2*B*). One variant (C67R) was associated with low expression with no associated paired subject serum level measurement. Encouragingly, there were no discrepancies between subject serum level and *in vitro* expression/secretion analysis in this study, with all other variants identified with a normal or unknown FI serum level.

**Figure 3:**
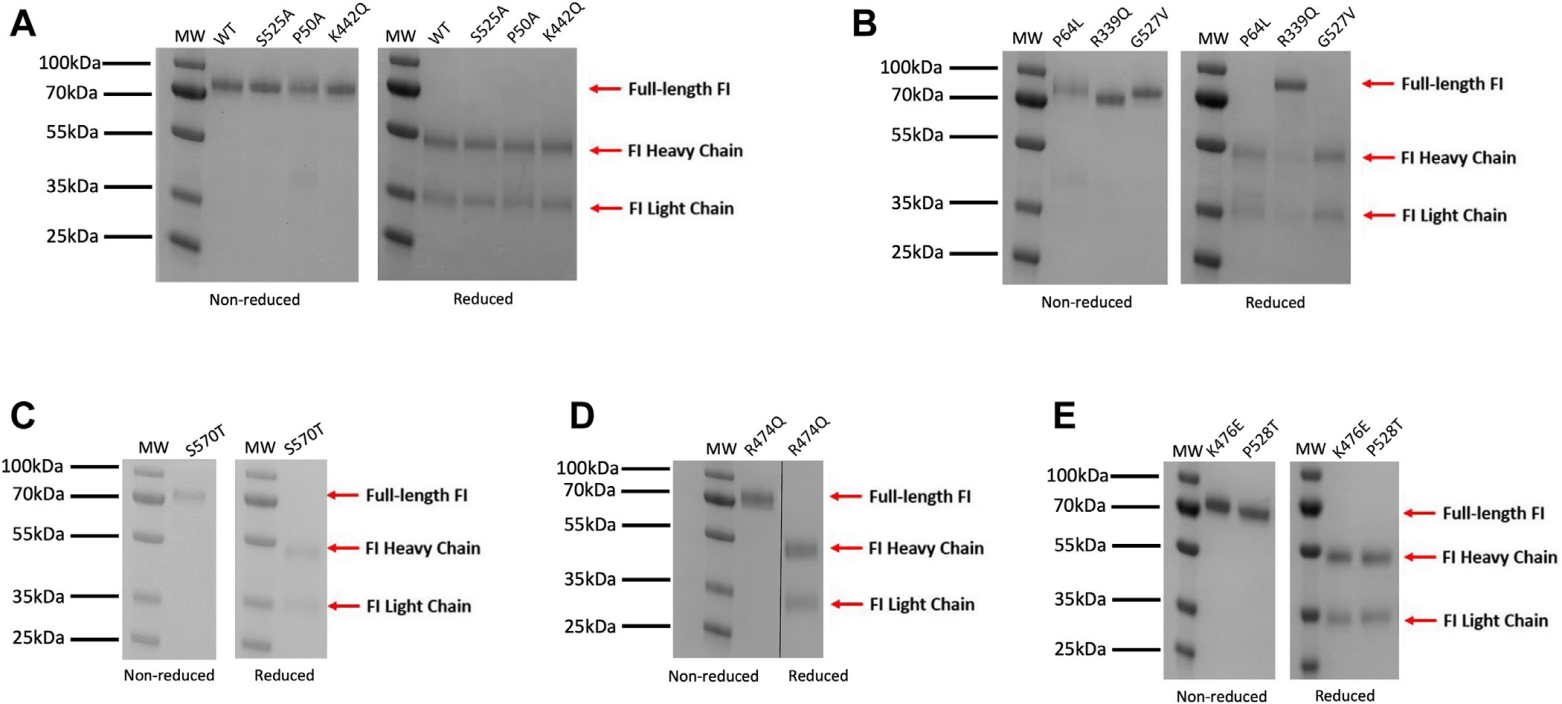
**SDS-PAGE for purified FI variants.** Purified FI variants and WT recombinant FI were subjected to standard SDS-PAGE (*A*–*E*). Coomassie blue-stained SDS-PAGE gels show ∼1 μg of purified FI under nonreducing (*left* gel *A*–*E*) or reducing conditions (*right* gel *A*–*E*) for each variant that could be made and the WT. The heavy chain of factor I is represented by the band at 50 kDa, whereas the light chain is represented by the band at 38 kDa. Splicing sites for removal of irrelevant lanes are marked by a *black line*. Ladder size: 10 to 250 kDa. MW, molecular weight.

SDS-PAGE were run for all purified variants (Fig. 3, *A*–*E*) revealing normal processing with no impurities or presence of the full length 88 kDa band in reducing conditions for any variant preparations apart from R339Q (Fig. 3*B*), which was expected due to mutation of the furin cleavage site in the RRKR linker.

### Functional assessment of FI variants by fluid phase cofactor assays of C3b cleavage

**Figure 4:**
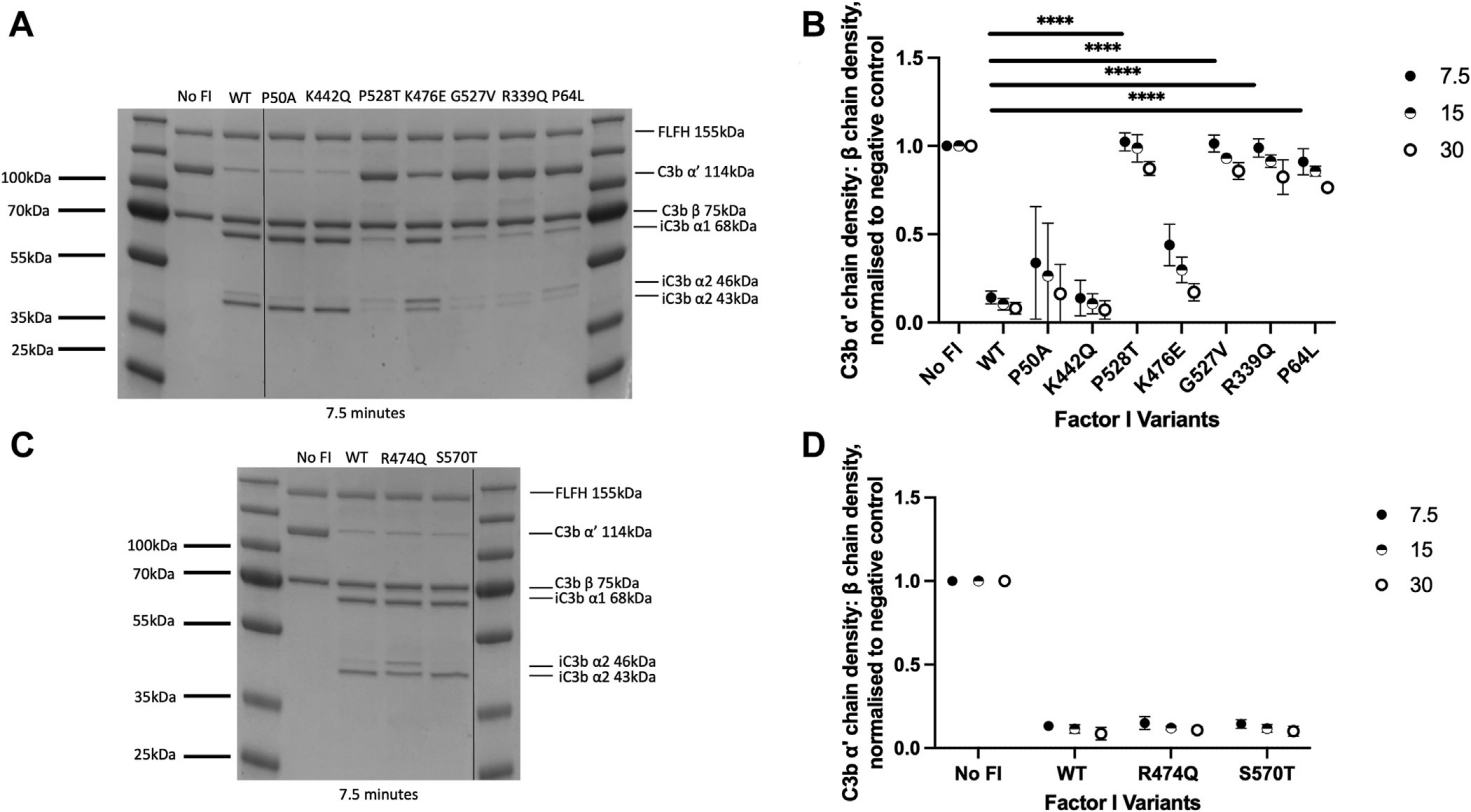
**Evaluation of fluid phase C3b cofactor activity of FI variants by SDS-PAGE and kinetic analysis.***A* and *C*, after incubation of each FI variant with C3b and FH, SDS-PAGE was utilized to demonstrate proteolytic activity over a range of time points (7.5–30 min; 7.5 min shown here. 15- and 30-min time points are shown in Fig. S1). FI enzymatic activity was assessed by monitoring the loss of the α′ band of C3b at 114 kDa and the generation of the α^1^ and α^2^ chains of iC3b. Splicing sites for removal of irrelevant lanes are marked by a *black line*. *B* and *D*, the remaining density of the C3b α′ chain (*Y*-axis) is plotted after incubation of each FI variant with C3b and FH for 7.5, 15, and 30 min at 37 °C. The density of the α′ chain band is normalized to the β chain band density of the same lane. The resulting figure is further normalized to a negative control lacking FI, yielding a proportion of α′ chain remaining in comparison to the no-FI control (no FI = 1; all α′ chain remaining). The normalized α′ chain remaining for each variant is provided as the mean (N = 3 for all variants apart from G527V (N = 2)) with SD presented as error bars and significance tested at 7.5 min using One-way ANOVA and Dunnett’s multiple comparisons test. ∗*p* < 0.05, ∗∗*p* < 0.01, ∗∗∗*p* < 0.001, ∗∗∗∗*p* < 0.0001.

To test the proteolytic activity of purified FI variants in the fluid phase for the AP central activating component, C3b, standard fluid phase cofactor assays were run using factor H as a cofactor. C3b breakdown was demonstrated by SDS-PAGE (Figs. 4, *A* and *C* and S1) and densitometry analysis was used to quantify α′ chain breakdown for each variant at three time points (Fig. 4, *B* and *D*). In this assay, K442Q, R474Q, and S570T were similar to WT in function, measured by cleavage and loss of the 114 kDa α′ chain of C3b. The C3b proteolytic activity of P50A was highly variable, with SD overlapping that of WT. K476E had somewhat reduced function compared to WT although this was not significant (*p* = 0.06), while P64L, R339Q, G527V, and P528T were all almost entirely abrogated in C3b cleavage function (all *p* < 0.0001). Notably, while normal in breakdown of the C3b α′ chain, R474Q appeared slower to perform the second clip to the 43 kDa chain of iC3b, with comparably more of the 46 kDa band present *versus* WT. Further description of C3b cleavage pattern and chains is shown in Fig. S3*A* and Figure 5*B* in Hallam *et al.* (27).

**Figure 5:**
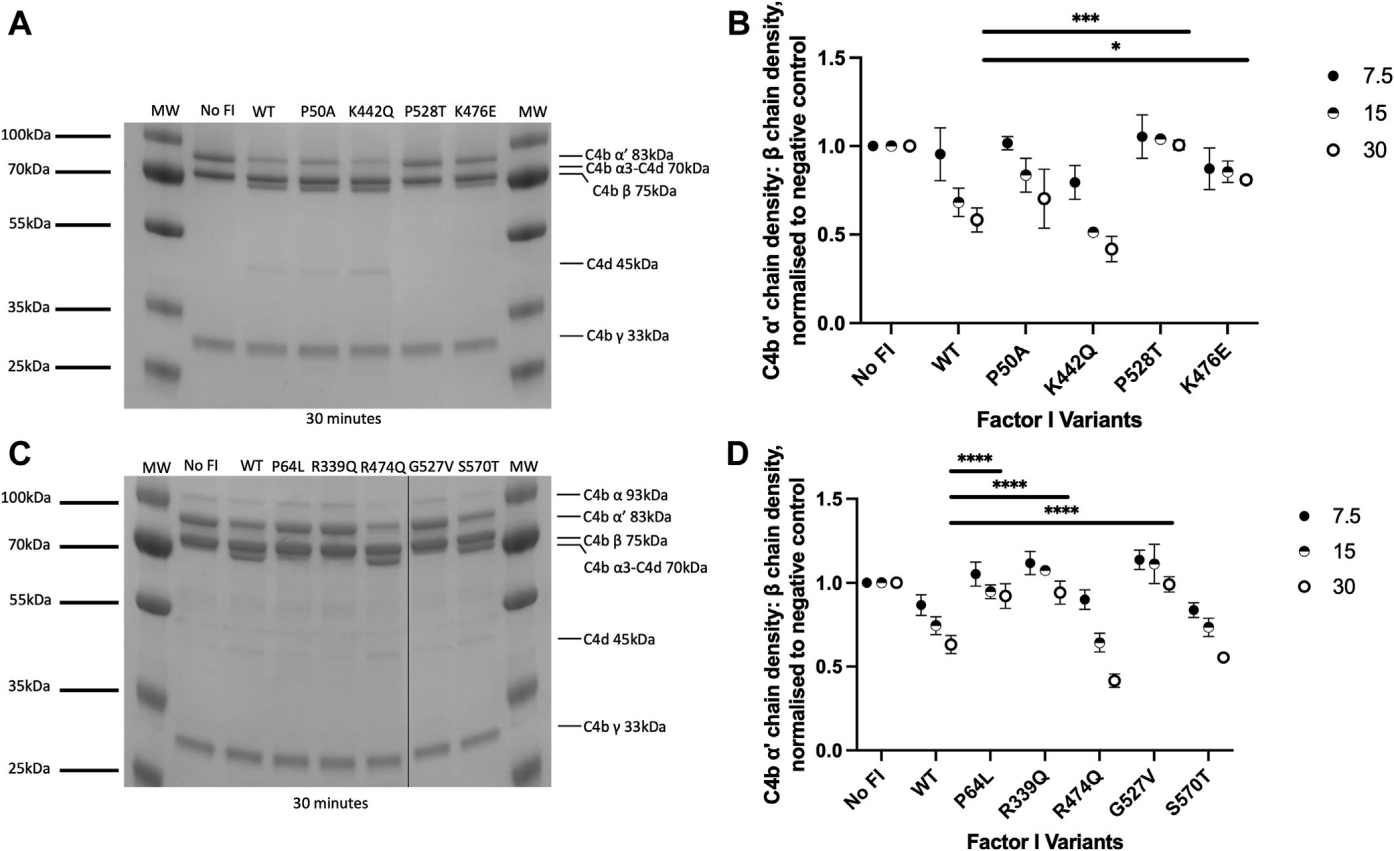
**Evaluation of C4b cofactor activity of FI variants by SDS-PAGE and kinetic analysis.***A* and *C*, after incubation of each FI variant with C4b and C4BP, SDS-PAGE was utilized to demonstrate proteolytic activity over a range of time points (7.5–30 min; 30 min shown here. 7.5 and 15 -min time points are shown in Fig. S2). FI enzymatic activity was assessed by monitoring the loss of the α′ band of C4b (83 kDa) and the generation of the C4d band (45 kDa). Splicing sites for removal of irrelevant lanes are marked by a *black line*. *B* and *D*, the remaining density of the C4b α′ chain (*Y*-axis) is plotted after incubation of each FI variant with C4b and FH for 7.5, 15, and 30 min at 37 °C. The density of the α′ chain band is normalized to the β chain band density of the same lane. The resulting figure is further normalized to a negative control lacking FI, yielding a proportion of α′ chain remaining in comparison to the no-FI control (no FI = 1; all C4b α′ chain remaining). The normalized α′ chain remaining for each variant is provided as the mean (n = 3 for all variants) with SD presented as error bars and significance tested at 30 min using one-way ANOVA and Dunnett’s multiple comparisons test. ∗*p* < 0.05, ∗∗*p* < 0.01, ∗∗∗*p* < 0.001, ∗∗∗∗*p* < 0.0001. C4BP, C4b binding protein.

### Functional assessment of FI variants by fluid phase cofactor assays of C4b cleavage

To test the proteolytic activity of FI variants in the fluid phase for the CP activating component, C4b, fluid phase cofactor assays were run using C4b binding protein (C4BP), as cofactor with an SDS-PAGE visualization of breakdown (Figs. 5, *A* and *C* and S2) and densitometry analysis for quantification (Fig. 5, *B* and *D*). As measured by loss of the α′ chain of C4b (83 kDa) after 30 min incubation with FI and C4BP, variants P50A, K442Q, R474Q, and S570T were shown to be either similar to or more active (R474Q) than the WT FI protein. Conversely, P64L (*p* < 0.0001), P528T (*p* < 0.001), K476E (*p* < 0.05), R339Q (*p* < 0.0001), and G527V (*p* < 0.0001) were reduced in C4b cleavage activity, with minimal C4b α‘ chain breakdown observed for these variants. These findings were generally concordant with fluid phase C3b cleavage data. Fig. S3*B* provides further description of C4b cleavage pattern and chains.

### Functional assessment of FI variants by bead-based functional assay of C3b cleavage

**Figure 6:**
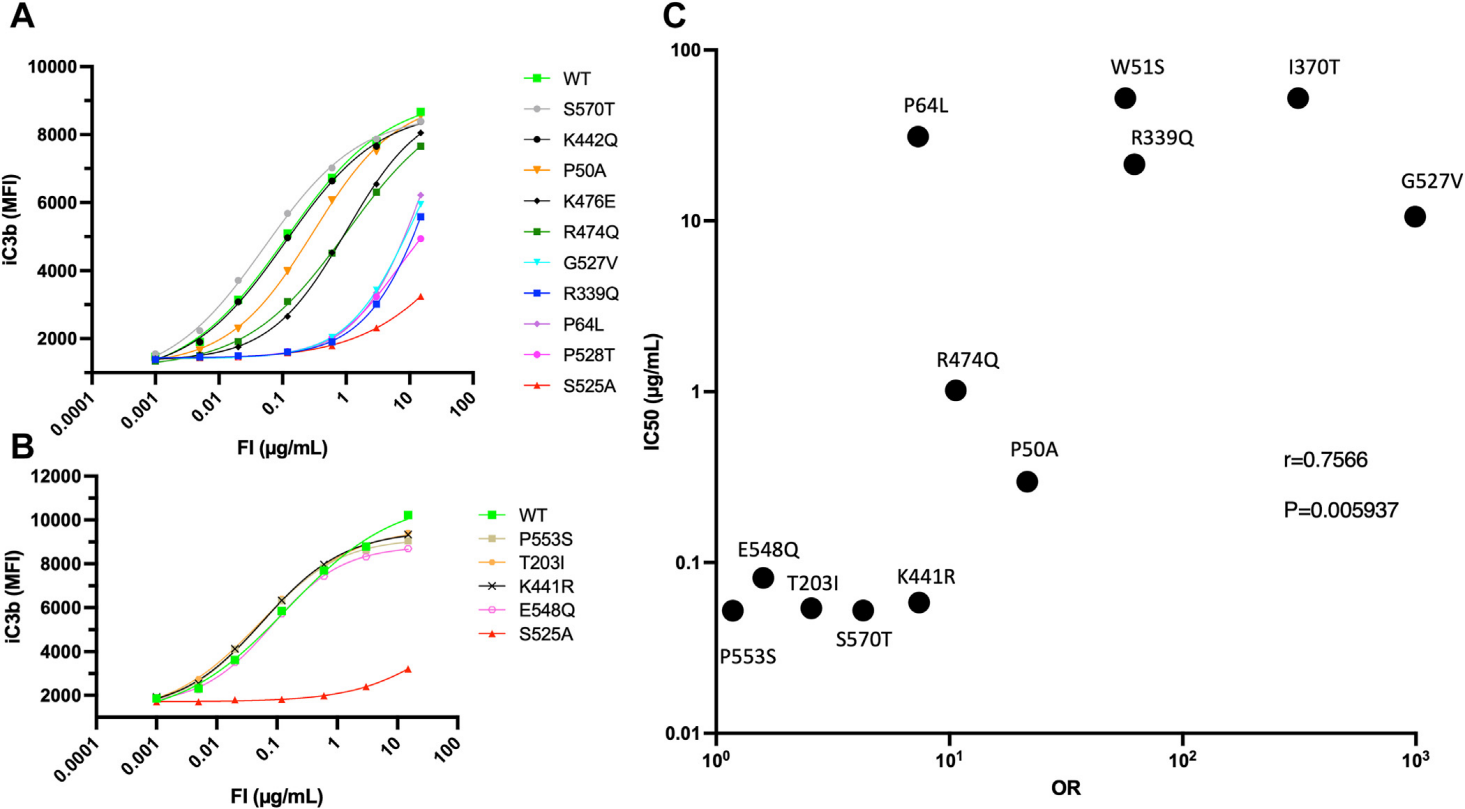
**Analysis of bead-based C3b cleavage IC50s for FI variants.***A*, bead-based functional assay (BBFA) of C3b cleavage. Each FI RV was titrated 1:4 into C3b-coated beads with excess FH and incubated for 1 h to allow cleavage of C3b by each FI variant. Function is directly proportional to signal where increasing MFI represents more iC3b formation. Four parameter logistic regression curves are shown by lines (WT: *light green*; P50A: *orange* P64L: *purple*; R339Q: *blue*; K442Q: *black* with *circles*; R474Q: *dark green*; K476E: *black* with *diamonds*; S525A: *red*; G527V: *cyan*; P528T: *pink*; S570T: *gray*). Each point shows the median fluorescence intensity (MFI) of a minimum of 1000 beads for each concentration of FI variant (μg/ml). Assay representative of three independent repeats for all variants apart from R474Q with two repeats. *B*, bead-based functional assay (BBFA) of C3b cleavage for T203I (*orange*), K441R (*black*), E548Q (*pink*), P553S (*gold*), compared to WT (*green*) and S525A (*red*). *C*, correlation analysis of BBFA IC50s *versus* odds ratio. Pairwise correlation analysis was carried out by plotting the OR (*X*-axis) and IC50 from the BBFA (*Y*-axis) for each FI variant and the Spearman correlation test was performed. An r value of 0.74 (*p* = 0.0059) suggests a moderate positive correlation between the two values (∗∗*p* < 0.01). RV, rare variants.

To complement the fluid phase analysis and to confirm each FI variant’s enzymatic function for C3b cleavage with increased confidence, a flow cytometric BBFA was utilized. In this assay, an antibody against an iC3b neoepitope was used such that a larger fluorescent signal, and a lower IC50, was expected on increased FI functional activity. Examining the curves in Figure 6*A*, and comparing IC50s from three independent repeats of the BBFA, the characterized FI variants could be defined into three distinct functional groups, those that are substantially reduced in function (IC50 (μg/ml) S525A: 52.24, P528T: 5.726, G527V: 10.568, R339Q: 21.35, P64L: 31.098), those that have a modest functional deficit (IC50 (μg/ml) R474Q: 1.019, K476E: 0.883, and to a lesser extent P50A: 0.297), and those with a function similar to that of WT FI protein (IC50 (μg/ml) K422Q: 0.107, S570T: 0.052, WT: 0.096). One notable deviation from the fluid phase assay is R474Q, which exhibited normal breakdown of the C3b alpha’ chain in that assay, however, this may be an artefact of the detection method given the iC3b neo-epitope may be exposed only on cleavage of C3f and not after the first clip of FI at R1020 only. In Figure 6*B* an additional four variants analyzed only by BBFA were shown to be similar to the WT FI protein in function, (IC50 μg/ml T203I: 0.054, K441R: 0.058, E548Q: 0.081 and P553S: 0.052, *versus* WT: 0.1 and S525A: 48).

### CADD score assessment of *CFI* variants

Predicting functional consequences of *CFI* RVs *in silico* has proved challenging; however, a recent tool called combined annotation dependent depletion (CADD) provides a multimodal approach to assign variant pathogenicity with greater accuracy (52). CADD produces standardized, genome-wide, variant scoring metrics that incorporates the weighted results of widely used *in silico* pathogenicity prediction tools (SIFT, PolyPhen) (53, 54) genomic annotation sources (ENCODE) (55) to produce a score expressed as a measure of deleteriousness (selection pressure bias) (52). A high CADD score represents variants that are not stabilized by selection, and which are more often disease-causing than expected by random chance.

CADD scores were generated for each FI variant (Table 2) and a further comparison of CADD to REVEL (56, 57) scores can be found in Table S1. All variants apart from the benign K442Q (CADD: 15.45) and K441R (CADD: 6.753) were associated with a CADD score of between 20 to 30, suggesting deleteriousness. This was not unexpected due to the selection process targeting those amino acid changes most likely to affect AP regulatory TMC binding or function. Moreover, three variants that resulted in little to no secreted protein (W51S, C67R, and I370T) were associated with a high CADD score of between 25 to 30, suggesting a greater likelihood these proteins would misfold or result in reduced expression and therefore result in the high-risk haploinsufficiency phenotype.

**Table 2:** Summary of CADD scores for all tested *CFI* RVs

Protein change	DNA position	Reference allele	Alternative allele	CADD (PHRED)	RawScore
S570T	109740936	C	G	24.3	3.526528
P553S	109740988	G	A	20.3	2.071278
E548Q	109741003	C	G	24.7	3.629463
P528T	109741063	G	T	25.7	3.868586
G527V	109741065	C	A	26.4	4.00859
S525A	109741072	A	C	25.5	3.823122
K476E	109746225	T	G	24.1	3.452032
R474Q	109746230	C	T	24.2	3.485108
K442Q	109746327	T	G	15.45	1.380392
K441R	109746329	T	C	6.753	0.413095
I370T	109749257	A	G	25.2	3.763436
R339Q	109749527	C	T	23.1	3.067982
T203I	109761567	G	A	12.48	0.943939
C67R	109766683	A	G	26.6	4.035458
P64L	109766691	G	A	26.3	3.978049
W51S	109766730	C	G	25.9	3.917961
P50A	109766734	G	C	23.5	3.254155

Two CADD outputs are described; CADD (PHRED) is a scaled score ranging from one to 99, based on the rank of each variant relative to all possible 8.6 billion substitutions in the human reference genome. RawScore is the raw value, where higher values indicating that a variant is more likely to be simulated (or “not observed”) and therefore more likely to have deleterious effects.

### Correlation analyses of FI variant *in vitro* functional characterization with measures of increased disease odds and *in silico* deleteriousness/pathogenicity

The results from *in vitro* functional characterization (IC50 generated by BBFA assay), magnitude of genetic association of *CFI* RV with increased AMD risk (OR), and *in silico* prediction of variant consequence (CADD score) are summarizedin Table 3. Three distinct pairwise correlation analyses were performed (OR *versus* IC50; OR *versus* CADD; and CADD *versus* IC50). Significant positive correlations were identified between the OR and the BBFA IC50 of variants (r = 0.76; *p* = 0.0059; Fig. 6*C*). In addition, the BBFA IC50s and CADD scores exhibited a correlation that remained positive but not significantly so (r = 0.43; *p* = 0.16; Fig. S3). However, correlation was identified between CADD scores and ORs (r = 0.71; *p* = 0.002; Fig. S4). Overall, considering the correlation between the IC50s and ORs of variants was the most positive correlation suggests that full functional characterization of FI’s enzymatic activity for C3b and C4b should remain the gold standard for an accurate understanding of the deleteriousness of rare genetic variants in *CFI*.

**Table 3:** Comparison of *in silico* and *in-vitro* characterization scores for CFI RVs that underwent functional investigation

FI variant amino acid change	CADD (PHRED)	OR	IC50	Classification
WT	NA	NA	0.096	NA
P553S	20.3	1.178	0.052	Benign
S570T	24.3	4.269	0.052	Benign
T203I	12.48	2.558	0.054	Benign
K441R	6.753	7.401	0.058	Benign
E548Q	24.7	1.591	0.081	Benign
K442Q (designed variant)	15.45		0.107	NA
P50A	23.5	21.560	0.297	Type II
K476E	24.1	NA	0.883	Type II
R474Q	24.2	10.650	1.019	Type I/II^a^
P528T	25.7	NA	5.726	Type II
G527V	26.4	993.900	10.568	Type II
R339Q	23.1	62.060	21.350	Type II
P64L	26.3	7.325	31.098	Type II
S525A (designed variant)	25.5	NA	52.240	NA
I370T	25.2	312.900	52.240^b^	Type I
C67R	26.6	NA	52.240^b^	Type I
W51S	25.9	56.760	52.240^b^	Type I

For each *CFI* RV, the *in silico* CADD (PHRED) score for variant deleteriousness, level of increased odds of GA disease (measured in SCOPE, OR; odds ratio), and *in vitro* function using IC50 as a quantitative readout from a bead-based functional assay (BBFA), are provided. IC50s are expressed as mean of three independent repeats removing outliers or noncalculable results.

Abbreviations: CADD, combined annotation dependent depletion; NA, not applicable; OR, odds ratio.

a R474Q resulted in reduced expression and reduced function and serum level.

b non-secreted variants assigned as 95%ile of IC50 for other genotypes.

### Final functional categorization of CFI RVs in SCOPE

To complete the analysis, variants in SCOPE were classified by the characterization presented herein, including our internal serum measurement, in addition to externally published data reviewed in Table 3 of Hallam *et al.* (27). As such, 3.2% of subjects carried *CFI* RVs classified as “type 1” (low FI levels; “haploinsufficient”), 0.6% as “type 2” (normal FI levels but dysfunctional protein), and 2.6% as benign (normal levels and function), leaving 0.6% carrying a variant of uncertain significance (Table S2). Of note, variants P553S, K441R, G261D, and R406H make up all but nine (of 88) benign carrier genotypes. Moreover, the type I G119R variant is carried by 1% of SCOPE GA cohort.

## Discussion

This study describes the findings from extensive *in vitro* functional characterization of 15 *CFI* RVs identified in GA subjects who were being assessed for participation in the SCOPE natural history study. Of these, 11 variants predicted to alter function were characterized using three assays assessing C3b and C4b cleavage, and transient transfection expression tests, whilefour variants of known or predicted minimal consequence were characterized by a single sensitive assay of C3b cleavage (the BBFA). The findings presented bolster our knowledge regarding *CFI* RV genotypes. For each variant, the resulting functional consequence was compared against magnitude of disease-associated odds, and *in silico* CADD prediction for measuring variant deleteriousness or pathogenicity, revealing that pure protein *in vitro* analyses remains the most informative for understanding genotype/phenotype relationships for *CFI* RV.

One variant, S570T, had no dysfunction across all assays, suggesting complete expression and fully functional cleavage of both C3b and C4b with FH and C4BP as respective cofactors. In keeping with this, S570T has previously been identified in an AMD cohort and was associated with normal circulating levels and function (40, 58). The topology of S570 at the C terminus of FI, and the relatively minor side chain property alteration of Ser to Thr could explain this finding. K442Q is reported herein as a benign designed variant, similar to K441R which was characterized previously as benign by multiple assays (49). As predicted, T203I, K441R, E548Q, and P553S had little or no effect on function by a single assay, the BBFA, and have all been reported associated with normal FI levels previously (27, 36, 37), and therefore were categorized as benign. Investigation by 3D modeling revealed that K441, K442 Fig. S5*A*), P553 (Fig. S5*B*), and T203 (Fig. S5*C*) are at surfaces of the FI HC or LC with no interaction with C3b and only K441 interacting with N136 of FH. S570 is found at a C-terminal loop that sits between the HC and LC (Fig. S5*A*). Meanwhile, of interest, E548 is in close proximity with the surface of C3b near P1301 (Fig. S5*B*). Whether any ionic interaction or salt bridge would form between E548 and P1301 is unclear however given that E548Q did not affect function of FI and that PyMOL modeling does not predict this suggests not.

P50A has been reported previously in several cohorts, and in multiple disease states including aHUS and AMD (37, 59); as a relatively more common (but still rare) variant of particular interest. P50A has previously been associated with a mix of normal or reduced antigenic level, and normal or reduced function, making categorization challenging (37, 39, 40, 58, 59, 60, 61). Alteration of a HC binding site hindering detection antibody binding may explain previous variability in classification of P50A as type I (62). Herein, we describe P50A associated with exclusively normal levels and function that was varied but not significantly different to WT in fluid phase assays. However, the more sensitive BBFA (Fig. 6*A*) reveals a ∼3-fold reduction in function compared to WT [IC50 0.3 *versus* 0.1 μg/ml] which could conceivably result in chronic low-level increase in complement activation contributing to disease (OR 21.56). The P50 side chain is buried within the FI LC and possibly important to the structure of the protein (Fig. S6*C*).

R474Q has been identified in aHUS and AMD, where it was reported with normal secretion but with reduced C3b cleavage function (40, 58, 63, 64). R474Q was the variant with the largest distinction between fluid phase assays and the BBFA in this study. However, in studying the fluid phase C3b assay gels in Figure 4*C*, differential kinetics can be observed with more of the first clip product (α^2^ 46 kDa) and less second clip generated (α^2^ 43 kDa) than the WT, despite similar breakdown of the C3b α’ (114 kDa) chain. We hypothesize therefore that reduced iC3b detection signal in the BBFA is due to reduced binding affinity of the neo-antibody to iC3b1 (46 kDa α^2^, C3f remaining in-tact) given that the major C3 conformational change occurs after the second cleavage and release of C3f (generating the α^2^ 43 kDa chain and iC3b) (65). This finding is reproduced by a second study wherein an iC3b ELISA is used as a read-out of function, and reduced function is exhibited (58). Whether or not the variant is deleterious depends on whether the iC3b1 intermediate molecule can amplify the AP, but we hypothesize it would not form an active C3 convertase due to conformational changes *versus* uncleaved C3b. In keeping, Xue *et al.* suggested an “increased flexibility or stretch of amino acids” between residues 1304 to ∼1323 after the first clip (65). However, one study did report reduced C3b cleavage in serum without requirement for a neo-epitope (63) Structurally, the R474 side chain interacts with the surface of C3b at K1306, mutation to which may perturb binding of FI to C3b in the C3f region of the CUB domain fitting with altered second clip function (Fig. S6*A*). An additional confounding aspect for R474Q characterization is that, we observed a low serum FI level in one subject carrier and reduced expression/secretion upon transient transfection *versus* WT, despite previous publication reporting it being associated with normal secretion. Further serum measurements would help build confidence in classifying this variant as type I.

K476E is a so far an unreported type II variant shown to be of normal antigenic serum level, somewhat reduced function in fluid phase assays (*p* < 0.05 for C4b cleavage, *p* = 0.06 for C3b cleavage) and a ∼1 log functional reduction by BBFA for C3b breakdown [IC50 0.88 *versus* 0.1 for WT]. K476E is located at the surface close to but not directly interacting with the FH CCP3 and C3b CUB domain binding interface (Fig. S6*A*), the swapping of a positive to negatively charged amino acid at this site could conceivably alter salt bridge formation within the AP regulatory TMC.

Four variants were categorized as highly deleterious type II secreted variants by this study (P64L, R339Q, G527V, and P528T). R339Q is unprocessed due to the mutation of the RRKR furin cleavage site that links the HC and LC of FI, as described previously (58). R339Q FI is likely inactive due to limited flexibility between the HC and LC inhibiting allosteric rearrangement of trypsin-like internal loops required for C3b cleavage. P64L has been reported previously in AMD (37, 40, 66), aHUS (67), and complete FI deficiency (68), with ∼50% reduced secretion (40) and low serum level (37, 66), while in this study expression of P64L was ∼50% of the WT protein. Analysis of function shows near entire abrogation in C3b, C4b fluid phase assays (both *p* < 0.0001) and the C3b BBFA [IC50 31 μg/ml *versus* 0.1 for WT]. In the context of deficiency of factor I with cerebral inflammation syndrome, the variant was identified in compound heterozygosity with a destabilizing Q88K variant, and a 3D analysis revealed an FI protein destabilizing affect for both and a steric C3b binding hindrance for P64L (at V1658 of C3b) (68). Our 3D modeling analysis identified the P64 side chain proximal to an interaction site with macroglobulin domain one of C3b possibly centered around a C3b E1654 salt bridge to FI R80 (Fig. S6*B*). Alteration of a HC binding epitope for detection antibody may explain variability in type I classification of P64L. G527V and P528T are to our knowledge previously unreported *CFI* RVs, they both result in near complete abrogation of function in C3b and C4b fluid phase assays (all *p* > 0.0001) and had BBFA C3b IC50s ∼1.5 to 2 log higher than WT, at 10.7 and 5.7 μg/ml, respectively (WT IC50: 0.1 μg/ml). These variants sit proximal to the catalytic serine of the FI LC (Fig. S6*D*), likely perturbing formation of the oxyanion hole required for cleavage of the scissile bonds at R1303 and R1320 of the C3b CUB domain (69). It is also possible that internal misfolding resulted in lower secretion of G527V compared to WT FI, although this is uncorroborated by serum measurement.

Finally, we identified 3 novel variants that were not secreted (W51S, C67R, and I370T). Of these, W51S and I370T were validated by low serum level in one individual each in the SCOPE cohort. Furthermore, a similar W51C variant was previously reported associated with a low FI serum level in aHUS (63). Structurally, both W51 and I370 are buried within the HC and LC of FI, respectively (Fig. S7, *A* and *C*). Meanwhile, serum FI measurements were not obtained for C67R; however, this variant results in loss of a cysteine likely to cause misfolding due a perturbed disulphide interaction with an internal cysteine (Cys86) in the HC of FI (Fig. S7*B*) adding confidence to its classification as type I.

This study describes findings from a highly sensitive C3b BBFA, a novel bespoke method with a similar technique published only once previously (61). A limitation to our study is that we do not apply a similar method to analyze C4b; however, analysis of C4b cleavage is likely less consequential for disease. Moreover, a good level of similarity was observed when comparing C3b and C4b cleavage in fluid phase assays, and we broadly would predict translation to the BBFA.

One useful tool used in evaluating drug targets is whether a dose-response relationship may be established between the functionality of a gene encoding the target and the degree or severity of a phenotype which is relevant to the mechanism of action. This relationship provides evidence in humans that pharmacological modulation of the implicated gene has the potential to move the target phenotype in the direction of clinical benefit. One way of approaching this is to define an “allelic series” where a collection of variants with increasingly deleterious mutations are identified which lead to increasing large phenotypic effects (70). This form of evidence has been instrumental in development of statins (71) and cementing support for *TYK2* as target in the treatment of psoriasis (72). We constructed an allelic series for *CFI* RVs using results from this study and observed a significant positive correlation between level of *in vitro* dysfunction as measured by the BBFA of C3b cleavage and increased AMD disease ORs. There was no correlation between *in silico* CADD scores of *CFI* RV deleteriousness/pathogenicity with the respective AMD ORs.

These findings support the recommendation that pure protein assays should remain the gold standard for predicting the consequences of secreted type II *CFI* variants, over *in silico* tools. High CADD scores correlate strongly to known variant pathogenicity (73, 74); however, this *in silico* tool was not able to fully predict the level of *CFI* RV (dys)function observed in the *in vitro* assays testing pure protein. Despite this, the performance of *in silico* tools are expected to improve, especially with assistance from generative artificial intelligence programmes. For example, while currently challenging, efforts are being made toward the use of AlphaFold (https://alphafold.ebi.ac.uk/) (75, 76) version 2 for the prediction of misfolding, posttranslational modification and subsequent dysfunction of amino acid mutations which could be correlated to disease risk or clinical outcomes (77, 78). The output of artificial intelligence models for variant prediction will need confirming with expert assessments like pure protein assays and serum measurement until enough data have been generated to show a high level of concordance and build confidence for their use in the clinic.

Comprehensive functional characterization of different *CFI* RV genotypes is important for the field as it supports clinical development of complement-targeted therapies and helps clinical diagnosis of inherited complement disorders that have a genetic etiology.

Subretinal FI gene therapy GT005/PPY988 in GA at phase II was recently declared futile [Press release: Novartis AG; September 11, 2023, 2023: https://www.novartis.com/news/gt005-ppy988-development-program-geographic-atrophy]. That said, Adverum are pursuing potentially in-office intravitreal administration of *CFI*-AAV7m8 to treat GA [Press release: Adverum biotechnologies; May 18, 2023: https://investors.adverum.com/news/news-details/2023/Adverum-Biotechnologies-Introduces-an-Intravitreal-Gene-Therapy-Program-for-Geographic-Atrophy-and-Presents-Data-on-Its-Ocular-Gene-Therapy-Platform/default.aspx]. In addition, the success of complement inhibitors in slowing GA disease progression shown by recent Food and Drug Administration approval of C3 and C5 inhibitors (Syfovre; Apellis and Zimura; Iveric Bio, respectively), indicate the complement pathway remains a viable strategy for treating GA and characterizing complement-related variant function is important for understanding subpopulation architecture. Furthermore, comprehensive nontargeted genetic screens like whole exome sequencing offered by direct-to-consumer companies like 23andMe or Ancestry, have the potential to uncover a greater breadth of *CFI* RV genotypes that may require functional characterization to help determine whether they are clinically relevant. This is especially relevant in individuals with non-European ethnicities where there is a currently a lack of genetic data in advanced age-related macular degeneration, and this may have implications for clinical development and commercialization strategies of future personalized complement therapies in advanced age-related macular degeneration to avoid bias in healthcare access (38).

Out of 15 studied variants, only S570T exhibited no signs of dysfunction or reduced expression, with four more variants showing normal function albeit by BBFA only, the remaining ten variants exhibited reduced function or expression by at least one measurement. Moreover, the allelic series in Figure 6 revealing strong correlation between AMD OR and *CFI* deleteriousness adds credence to therapeutic modulation of *CFI* in AMD. However, a recent report by Seddon *et al.* revealed much more modest ORs for progression between early and late AMD (42) compared to previous studies comparing AMD and non-AMD (36, 37, 43).The finding that increasingly dysfunctional *CFI* RVs correlate more strongly with disease suggests that unchecked generation of C3b and its products contributes to AMD pathology. The mechanisms underpinning C3b′s contribution to AMD are thought to primarily rely on the recruitment and activation of immune cells in the retina and choroid through C3b/iC3b-CR3 interactions and the fueling of a proinflammatory microenvironment by C5a generation, well reviewed in (79, 80).

Overall, characterization of *CFI* RVs in this study could be used to guide future treatments for patients with disease in a personalized medicine approach. *CFI* RV categorizations should be added to a database and compendium of *CFI* variant analysis in disease (as in https://www.complement-db.org/home.php (81)), and the SCOPE study should be an example for future endeavors in genotyping/phenotyping for patient stratification in clinical trials.

## Experimental procedures

### Patient cohort

Subjects with a diagnosis of GA as determined by a retinal specialist were considered for entry into SCOPE natural history study (ClinicalTrials.gov identifier, NCT03894020), sponsored by Gyroscope Therapeutics, a Novartis company. Subjects were selected for *CFI* genotyping if they had unilateral or bilateral GA as determined by fundus autofluorescence and a reading performance of ≥40 letters by best corrected visual acuity. Subjects were excluded who had macular neovascularization or diabetic retinopathy, as determined by the principal investigator at the screening site. This study received institutional review board, ethics committee, and regulatory authority approval and written consent was provided by all subjects in accordance with the declaration of Helsinki, as described previously (38, 43) and in a site-dependent manner: United Kingdom (Medicines and Healthcare products Regulatory Agency, London, and North of Scotland Research Ethics Committee), United States of America (Oregon Health & Science University, Portland, The Johns Hopkins Medicine IRB, Baltimore, Wills Eye Hospital IRB, Philadelphia, Columbia University IRB, New York and Advarra), Australia (Therapeutic Goods Administration, Woden, Bellberry Human Research Ethics Committee, Eastwood), Germany (Ethikkommission der Medizinischen Fakulta¨t der Eberhard Karls Universita¨t und am Universita¨tsklinikum Tu¨bingen), France (Comite´ de protection des Personnes Sud Me´diterranne´e I–Marseille), The Netherlands (CMO Regio Arnhem-Nijmegen UMC St Radboud) and Spain (CEIC-Hospital Clinico San Carlos, Madrid) Poland (Komisja Bioetyczna przy Bydgoskiej Izbie Lekarskej, Komisja Bioetyczna przy Okregowej Izbie Lekarskiej w Lodzi, Komisja Bioetyczna Slaskiej Izby Lekarskiej w Katowicach, Office for Registration of Medicinal Products, Medical Devices and Biocidal Products, Warszawa).Patients defined their race or ethnicity as either White, Hispanic or Latino, American Indian or Alaskan Native, Asian, Black or African American, Native Hawaiian or Other Pacific Islander, other, unknown, or not reported. Approximately 98% self-identified as White.

### Genetic testing and statistical analysis

Saliva was obtained from subjects with GA under SCOPE eligibility assessment and extracted DNA was analyzed for *CFI* RV using targeted next generation sequencing as described previously (38, 43). Variant positions were provided to genome build hg19/GRCh37 coordinates, *CFI* transcript accession number NM_000204.5 and FI protein accession number NP_000195.2. Genotypes were annotated with minor allele frequencies from NFE in GnomAD (V4.0) (50). The term “rare” refers throughout to variants that are equal or less than 1% MAF in NFEs. Data were accessed through the gnomAD browser (http://gnomad.broadinstitute.org) and the term “observed Afs” was used as the value representing the count ratio of the actually detected minor alleles to reliably sequenced alleles. For each genotype, the MAF was calculated as the number of *CFI* RV alleles divided by total alleles in the respective dataset. The association of *CFI* RV with GA was calculated using Chi squared test, apart from those cohorts reporting frequency of <5, where we used Fisher’s exact test. OR confidence intervals were derived using Woolf logit interval calculation. Statistics was conducted using GraphPad PRISM (v10.1.2, https://www.graphpad.com/).

### Serum FI measurement

FI serum level measurement of SCOPE subject samples was performed by Eurofins BioPharma service using a fully validated FI sandwich ELISA (Hycult Biotech). The mean FI level in SCOPE was 19.1 μg/ml (SD ± 3.5), a low level cutoff threshold was set as 15.6 μg/ml, as previously published (43).

### CADD score annotation

CADD scores were calculated using an online tool at https://cadd.gs.washington.edu/snv (V1.7) and variant positions described according to genomic build GRCh38/hg38.

### Recombinant protein production and purification

Recombinant factor I was produced using previously optimized methodology in our lab (49). In brief, VectorBuilder Inc was used to design a WT polycistronic IRES vector with furin and the full *CFI* cDNA sequence (NM_000204.4). Further details are available at https://en.vectorbuilder.com/vector/VB171219-1127wqz.html. Site-directed mutagenesis was carried out to generate *CFI* variants (P50A, W51S, P64L, C67R, I370T, R339Q, K442Q, R474Q, K476E, G527V, P528T, and S570T). The primers for *CFI* site-directed mutagenesis are described in Table S3.

Transfection of HEK293T (Thermo Fisher Scientific) with mutated and WT cDNA was performed using JetPEI (Polyplus) using the manufacturer’s instructions and stable FI expressing clones were generated for functional analysis using hygromycin selection. Following standard HEK culture in multilayer flasks (Millicell HY Multilayer T-100 Flasks, Merck) supernatant was obtained for FI purification performed using NHS-activated HiTrap (Cytiva) affinity or Affi-Gel Hz immunoaffinity columns (Bio-Rad) coupled with OX21 (monoclonal antibody to human FI, Merck) and elution with 0.1 M glycine pH 2.7. Pure protein was buffer exchanged into PBS using PD-10 desalting columns (Cytiva) and stored at −80 °C prior to analysis.

For analysis of expression and secretion for FI variants *versus* WT, HEK293T were transiently transfected with the cDNA plasmid for each variant in 6-well plates, and supernatant was taken after 72 h of expression without the use of hygromycin for selection (N = 3 min of three wells for each variant, N = 10 for WT).

S525A FI was generated previously in our lab (49). Plasmid cDNA for T203I, K441R, E548Q, and P553S in the same *CFI*-IRES-Furin backbone was purchased directly from VectorBuilder Inc, and purification was carried out using the same method.

### SDS-PAGE

The purified FI proteins were run on 10 to 20% SDS-PAGE gels (Novex Tris-Glycine Mini Protein Gels, Thermo Fisher Scientific) to investigate purified protein size by Coomassie (InstantBlue, Abcam) staining in reduced (1% beta-mercaptoethanol) and non-reducing conditions. Each FI protein (∼1 μg) was run alongside a PageRuler Plus Pre-stained molecular weight marker (10–250 kDa) (Thermo Fisher Scientific) at 170v for 45 min. The same method was utilized for visualization of C3b and C4b breakdown for the fluid phase assays.

### ELISA

In order to screen for recombinant FI expression and secretion for each variant, 96 well-plates (Maxisorp, Thermo Fisher Scientific) were coated with 2 μg/ml of the polyclonal sheep anti-human FI antibody (LS-C147802, LSBio) in a coating buffer (16% 0.2 M sodium carbonate, 34% 0.2 M sodium bicarbonate in water, pH 9.6) and were incubated overnight at 4 °C. The next day, the plates were washed three times with phosphate-buffered saline (PBST) (0.1% Tween 20) using a Wellwash Microplate Washer (Thermo Fisher Scientific, 5165040) and then 100 μl of blocking buffer solution [1% bovine serum albumin (BSA) (Sigma-Aldrich, A7906)] in PBST, were added to each well. After a 2 h incubation in blocking buffer at room temperature (RT), the plates were washed three times with PBST. Fifty microliters of the samples and standard were added to the plate in triplicate and duplicate, respectively. The FI standard (A138, CompTech) was diluted to a final concentration of 1 μg/ml in blocking buffer before being serially diluted a minimum of 7 times 1:2 across the plate. The sample supernatant was also diluted 1:2 with blocking buffer and then added to the wells. After incubating the plates for 1 h at RT, they were washed again three times with PBST. The wells were then treated with 50 μl of the monoclonal antibody OX-21 (1 μg/ml in blocking solution, in-house) for 1 h at RT, and then washed three times with PBST. Afterward, the plates were incubated with 50 μl of horseradish peroxidase-conjugated donkey anti-mouse secondary Ab (715-035-150JIR, Stratech) (1:8000 in blocking buffer) for 30 min at RT. Before adding 100 μl of tetramethylbenzidine solution to each well, a final wash (3 x PBST) was performed. After incubating the plate on a plate shaker for 10 min at RT, the reaction was stopped by adding 100 μl of 1 M sulfuric acid. Using a Labtech LT-4500 MTP Microplate reader (Tecan), the absorbance was instantly measured at 450 nm. GraphPad Prism 9.5.0 was used to interpolate the concentration of FI in a sample from the standard curve. The log (concentration)-response standard curve was created using a four-parameter logistic regression model. The concentration for each variant was given as a % of the WT concentration (minimum N of three wells for each variant).

### Fluid phase cofactor assays

#### C3b

Fluid phase cofactor assays were performed to detect differences in serine protease activity between the FI variants and the WT FI. The C3b cofactor assays were used to evaluate the conversion of C3b to its inactive form, iC3b, in the presence of full-length Factor H as a cofactor. C3b was cleaved at Arg1303-04Ser (generating α2 46 kDa and α1 68 kDa) and Arg1320-21Ser (generating α2 43 kDa and releasing C3f) to demonstrate FI activity. Purified FI (75 ng) was added to 1 μl of C3b (CompTech, A114, 1 μg/μl) (1 μg) along with 0.25 μl of 1 μg/μl FH (250 ng) (CompTech, A137) and diluted to 15 μl in PBS for each tested FI variant at each time-point. A negative control of C3b (1 μg) and FH (250 ng) in 15 μl PBS was included for each assay. Each reaction mixture was incubated for 7.5, 15 and 30 min at 37 °C. At each time point, 5 μl of 4x reducing sample buffer was added to 15 μl of each test and negative control to stop the reaction. The products were then visualized using reducing conditions and Coomassie staining on a 10 to 20% SDS-PAGE gel. Three independent repeats were performed for each assay at each time-point for every variant (N = 3) apart from G527V (N = 2).

#### C4b

Fluid phase C4b cofactor assays were used to evaluate the conversion of C4b to the inactive form, C4d, in the presence of C4BP as a cofactor. After one cleavage, the breakdown of C4b α′ chain (83 kDa) produces an α3-C4d chain of ∼70 kDa, plus an α4 chain (13 kDa), and after two cleavages, C4d (45 kDa) is generated in addition to an α3 chain (25 kDa). Purified FI (18.8 ng) was added to 1.5 μl of C4b (CompTech, A108, 1 μg/μl) (1.5 μg) along with 0.25 μl of 1 μg/μl (250 ng) C4BP (CompTech, A109) and diluted to 15 μl in PBS for each FI protein tested at each time-point. A negative control of C4b (1.5 μg) and C4BP (250 ng) in 15 μl PBS was included for each assay. Each reaction mixture was incubated at 37 °C for 7, 15, and 30 min. Next, 15 μl of each test were added to 5 μl of 4x reducing sample buffer at each time point to quench the reaction. The products were then visualized using reducing conditions and Coomassie staining on a 10 to 20% SDS-PAGE gel. Three independent repeats were performed for each assay at each time-point for every variant (N = 3).

Densitometry analysis was carried out using Image Studio Lite (Licor) to measure the degradation of the C3b α′ chain and the C4b α′ chain before comparing the activity of each FI variant to the WT. In both assays, the α′ chain density was normalized to that of the β chain for each test, since the β chains of C3b and C4b remain intact and therefore function as a loading control. The normalized results were presented as the ratio of α′ chain remaining for each test *versus* the negative (no FI) control for each assay. The activity for each variant was then compared to the WT by standard Dunnett’s multiple comparisons test.

### Bead-based functional assay of C3b breakdown

Human C3b protein (A114, CompTech) was biotinylated using EZ-Link Melamide-PEG2-Biotin (Thermo Fisher Scientific, A39261) following the manufacturer’s protocol. Beads were prepared by coating 4.5 × 10^7^ beads/ml of M-270 Streptavidin Dynabeads (Thermo Fisher Scientific, 65305) with biotinylated human C3b (2.5–5 μg/ml) for 1 h at RT with continuous mixing. Next, C3b coated beads were plated in a flat-bottomed 96-well plate at a final concentration of 1 × 10^7^ beads/ml and washed using an automated plate washer with a flat magnet twice with PBS-0.05% Tween 20. Next, the beads were incubated with FI (WT or variant) titrated 1:4 8 times starting at 15 μg/ml final concentration, spiked with an excess (20 μg/ml) of complement factor H (A137, CompTech), and incubated at 37 °C shaking at 750 RPM for 1 h. Beads were washed as previous and incubated with a high salt buffer (1% BSA, 1M NaCl in PBS) at RT shaking for 20 min. The beads were washed and blocked in 5% BSA in Cell stain buffer (BioLegend, 420201) before staining with a murine monoclonal anti-iC3b antibody (Quidel, A209; used at 1:1000) shaking at RT for 30 min. The plate was washed as previously and incubated with chicken anti-mouse A488 secondary antibody (Thermo Fisher Scientific, A-21200, 1:100). The beads were washed and resuspended in 120 μl 0.5% BSA in PBS in a round-bottomed 96-well plate for flow cytometric analysis (FACSLyric, BD Biosciences). Immunostained beads were probed with a blue laser at 488 nm with 527/32 filters to measure A488 fluorescence intensity, gating was used to isolate single bead populations avoiding inclusion of bead aggregates in the analysis. Median fluorescence intensities were obtained and fitted using 4PL (4 parameter logistic regression model) curves using GraphPad Prism V9 to generate IC50s.

### Statistics

Functional assay data were analyzed using GraphPad Prism v9 (GraphPad Software, San Diego, CA; www.graphpad.com). Densitometry analysis of cofactor assays was carried out using Image studio V5.2 (LI-COR, https://www.licor.com/bio/). A one-way ANOVA and Dunnett’s multiple comparisons test was used to compare the activity of FI variants to the WT in fluid phase cofactor assays at 7.5 min or 30 min for C3b and C4b assays, respectively. For the BBFA, IC50s were calculated using a 4-parameter fit logistic regression curve and were presented as the mean of N of three independent repeats. Correlation analysis was performed by Spearman correlation test with a two-tailed *p* value. Statistically significant results are indicated by (∗), (∗∗), (∗∗∗) or (∗∗∗∗) and defined as ∗*p* < 0.05, ∗∗*p* < 0.01, ∗∗∗*p* < 0.001 and ∗∗∗∗*p* < 0.0001.

## Data availability

All data supporting this study are reported within this manuscript. Raw data are available from the corresponding author upon reasonable request.

## Supporting information

This article contains supporting information (49, 56, 57, 62, 65).

## Conflict of interest

D. K.: Gyroscope Therapeutics (consultancy, equity, grant income), Novartis (consultancy), Alexion Pharmaceuticals (consultancy), Apellis (consultancy); Sarepta (consultancy), Chemocentryx (Consultancy), Sobi (consultancy), Samsung (consultancy), Purespring (consultancy), Roche (consultancy); C. L. H: Q32 Bio (consultancy), Gyroscope Therapeutics (Consultancy), Novartis (employee), Ra Pharmaceuticals (Grant income), Biocryst (consultancy); K. J. M: Qualasept (consultancy), Freeline Therapeutics (consultancy), Catalyst Biosciences (grant income, consultancy), Idorsia Pharmaceuticals (grant income), Gemini Therapeutics (grant income, consultancy), Alexion Pharmaceuticals (grant income, consultancy); T. M. H.: Gyroscope Therapeutics, Novartis (employee); S. J. S.: Gyroscope Therapeutics, Novartis (employee); E. G.: Gyroscope Therapeutics, Novartis (employee); A. D.: Gyroscope Therapeutics, Novartis, Beacon Therapeutics (employee); A. V. J.: Gyroscope Therapeutics, Novartis (employee); A. L.: Gyroscope Therapeutics (consultancy, equity), Roche (consultancy), Apellis (consultancy), Novartis (consultancy).
