# Supplementary material for: Comprehensive functional characterization of complement factor I rare variant genotypes identified in the SCOPE geographic atrophy cohort
Source: J Biol Chem. 2024 Jun 7;300(7):107452. doi: 10.1016/j.jbc.2024.107452 (PMC11277764; doi:10.1016/j.jbc.2024.107452)
Supplement: Supporting Information [file mmc1.docx]

Comprehensive functional characterization of Complement factor I rare variant genotypes identified in the SCOPE Geographic Atrophy cohort

Thomas M Hallam^1*§^, Anneliza Andreadi^2,3§^, Scott J Sharp ^1^, Vicky Brocklebank ^2,3^ Emanuela Gardenal^1^, Anna Dreismann^1^, SCOPE Study GroupƗ, Andrew Lotery^5^, Kevin J Marchbank^2,3^, Claire L Harris^1,2^, Jones V Jones^1^, David Kavanagh^2,3,4*^

**Figure S1:** SDS-PAGE gels for fluid phase assays of C3b cleavage at 15- and 30-minute time points.

**Figure S2:** SDS-PAGE gels for fluid phase assays of C4b cleavage at 7.5- and 15-minute time points.

**Figure S3:** Cleavage of C4b and C3b by FI and its cofactors.

**Figure S4:** Correlation analysis of CADD scores vs Odds ratios and IC50s vs CADDs.

**Figure S5:** Three-dimensional modelling of Benign FI RVs.

**Figure S6:** Three-dimensional modelling of Type II dysfunctional FI RVs.

**Figure S7:** Three-dimensional modelling of Type I FI RVs.

**Table S1:** Comparison of combined annotation depletion dependent (CADD) score to REVEL score of variant deleteriousness.

**Table S2:** SCOPE cohort *CFI* genotypic characterization.

**Table S3:** Mutagenesis primers for point mutation generation within the *CFI* gene.

**
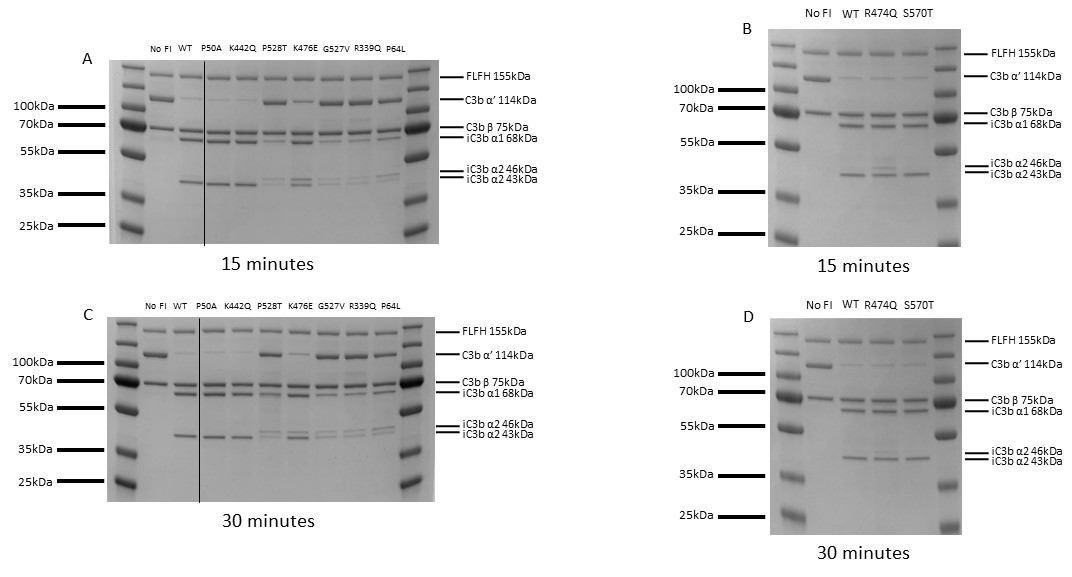
Figure S1:** SDS-PAGE gels for fluid phase assays of C3b cleavage at 15- and 30-minute time points (A-D). After incubation of each FI variant with C3b and FH, SDS-PAGE was utilized to demonstrate proteolytic activity over a range of time points (7.5 – 30 minutes; 15 (A,B) and 30 (C,D) minutes shown). FI enzymatic activity was assessed by monitoring the loss of the α’ band of C3b at 114kDa and the generation of the α^1^ and α^2^ chains of iC3b. Splicing sites for removal of an irrelevant lane are marked by a black line.

**
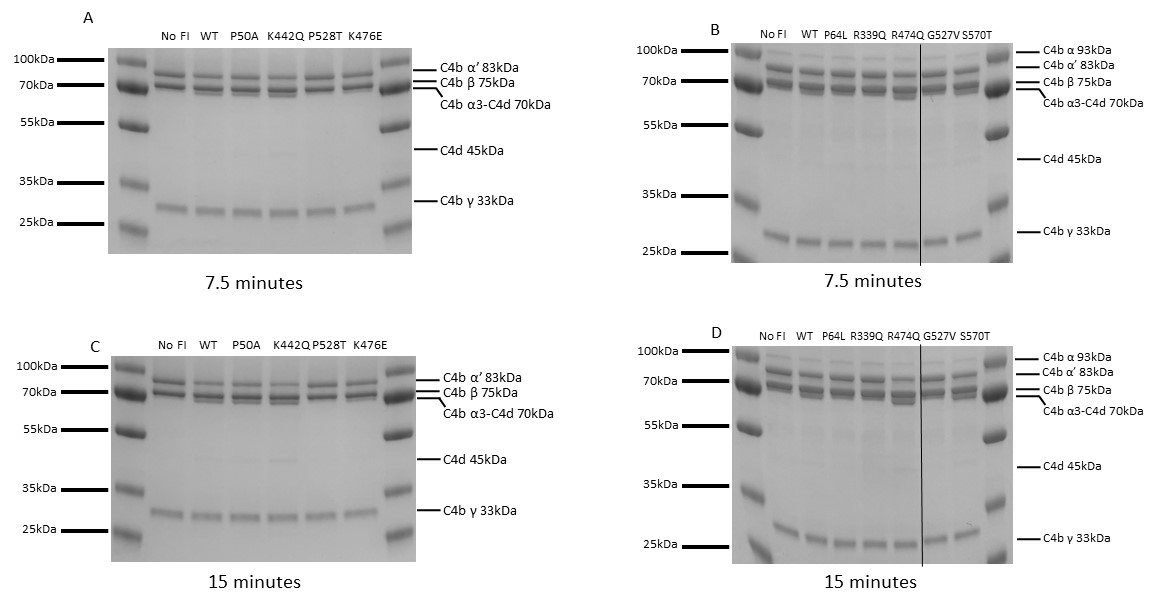
Figure S2:** SDS-PAGE gels for fluid phase assays of C4b cleavage at 7.5- and 15-minute time points (A-D). After incubation of each FI variant with C4b and C4BP, SDS-PAGE was utilized to demonstrate proteolytic activity over a range of time points (7.5 – 30 minutes; 7.5 (A,B) and 15 (C,D) minutes shown). FI enzymatic activity was assessed by monitoring the loss of the α’ band of C4b at 83kDa and the generation of the C4d band at 45kDa. Splicing sites for removal of an irrelevant lane are marked by a black line.

**
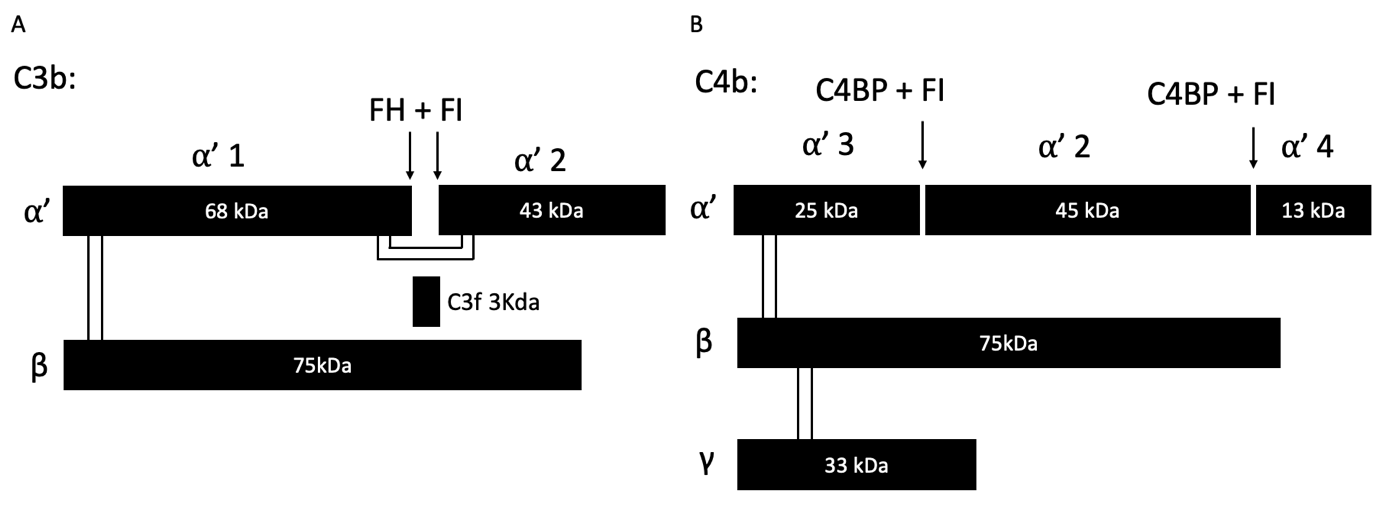
**

**Figure S3:** Cleavage of C4b and C3b by FI and its cofactors. A) Cleavage of C3b by FI and FH. The ⍺’ and β chains of C3b are displayed by black rectangles, and the cleavage sites of FI with FH as cofactor are indicated by arrows, meanwhile, double lines indicate disulphide bridges. B) Cleavage of C4b by FI and C4b binding protein (C4BP). The ⍺’, β, and γ chains of C4b are displayed by black rectangles, and the cleavage sites of FI with C4BP as cofactor are indicated by arrows, meanwhile, double lines indicate disulphide bridges.

**
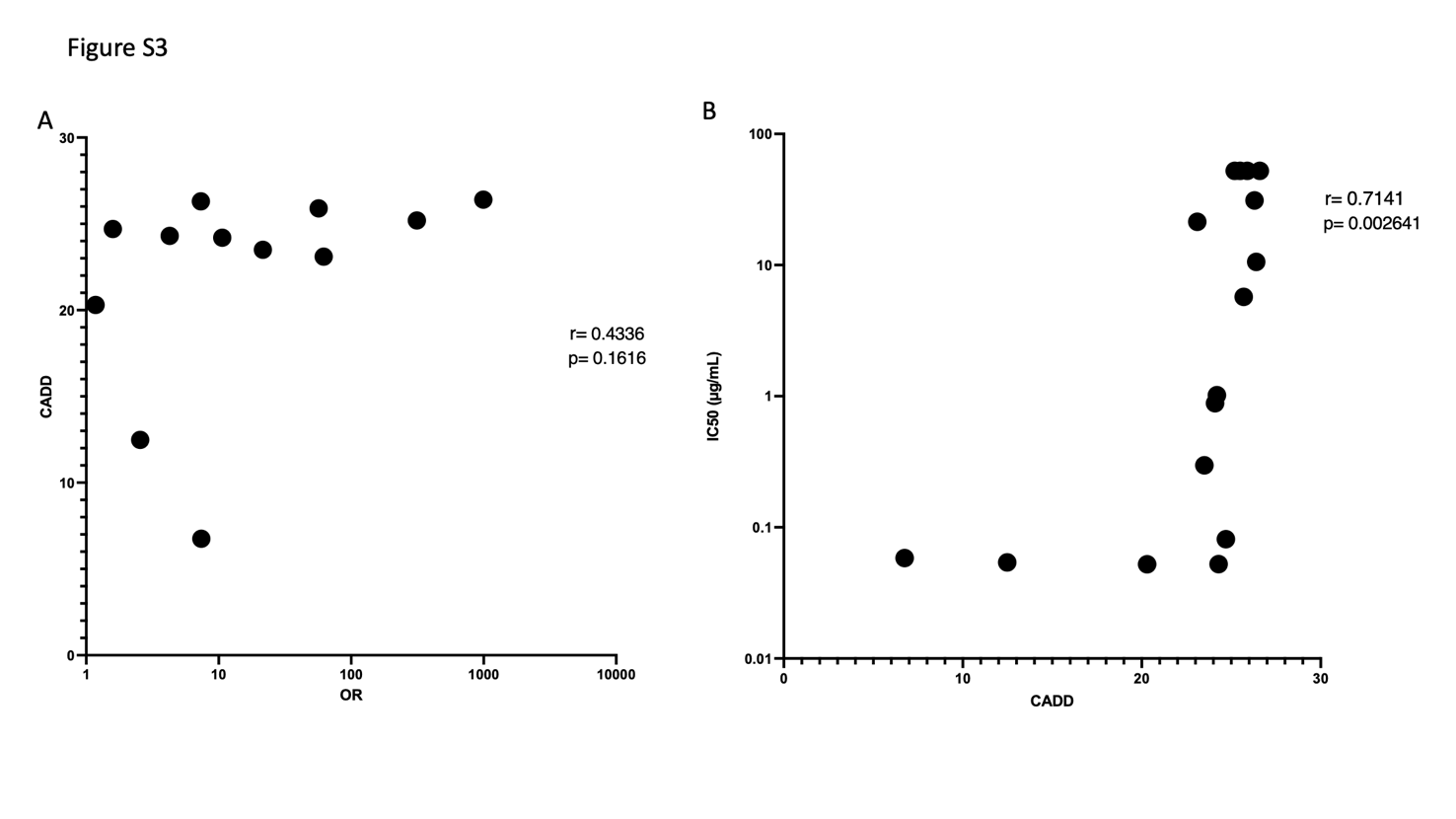
**

**Figure S4:** Correlation analysis of CADD scores vs Odds ratios (A) and IC50s vs CADDs (B). Pairwise correlation analysis was carried out by plotting the OR (X-axis) and CADD score (Y-axis) or CADD score (X-axis) vs IC50 from the BBFA (Y-axis) for each FI variant and the Spearman correlation test was performed. A positive r value of suggests a weak positive correlation for both pairwise tests but no significance was identified.


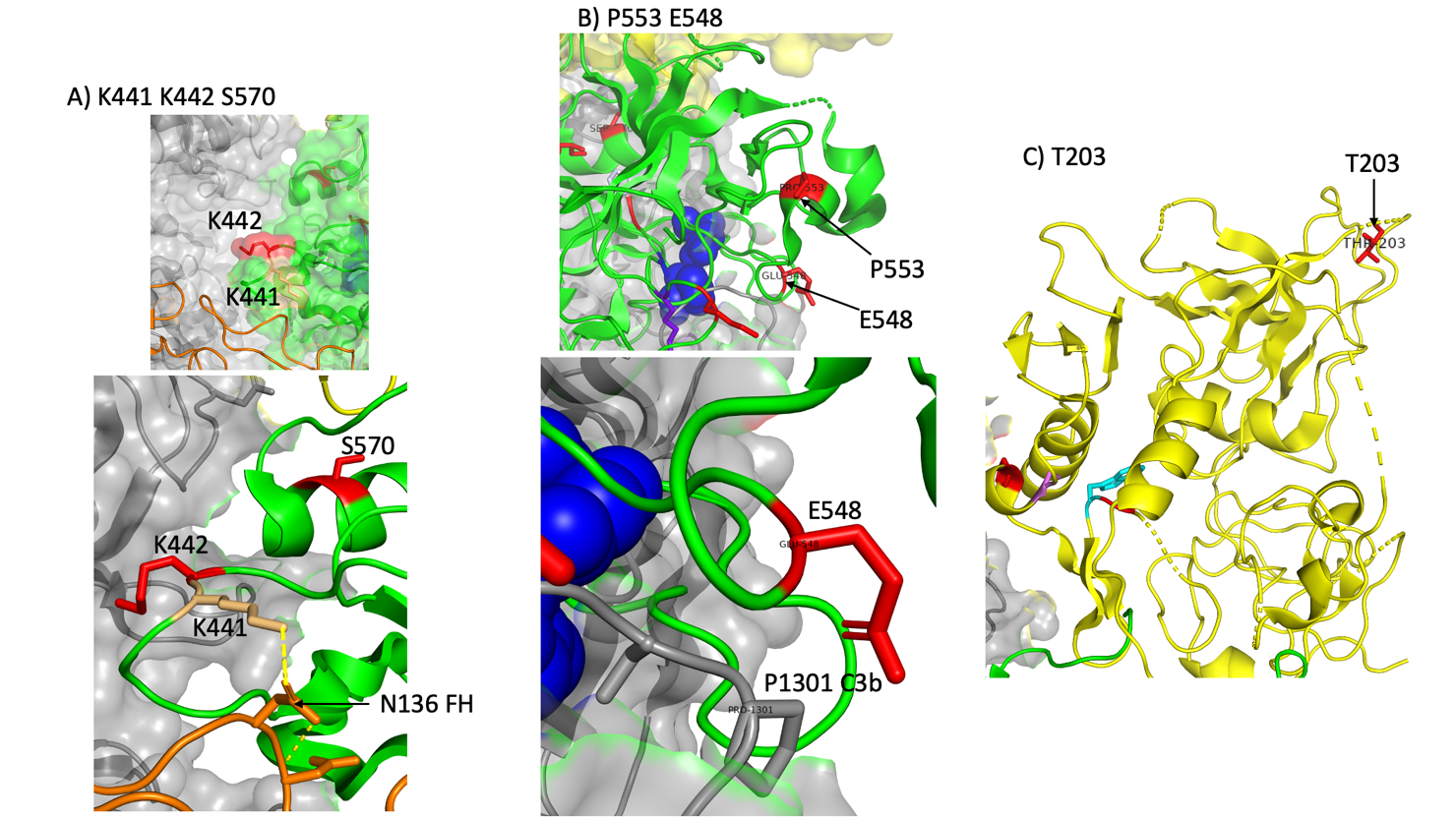


**Figure S5:** Three-dimensional modelling of Benign FI RVs. Benign FI RV side chains and their valence are shown by sticks in position within the 3D structure of the FI LC (green), HC (yellow) in complex with C3b (grey) and FH (orange) for A) K441, K442 and S570; B) E548Q and P553S; C) T203. Polar interactions where present between molecules are shown by yellow dashes. This modelling was performed in and images were produced using PyMOL^TM^ v2.5.4 (Schrodinger, LLC) and PDB structure 5o32 (1).

**
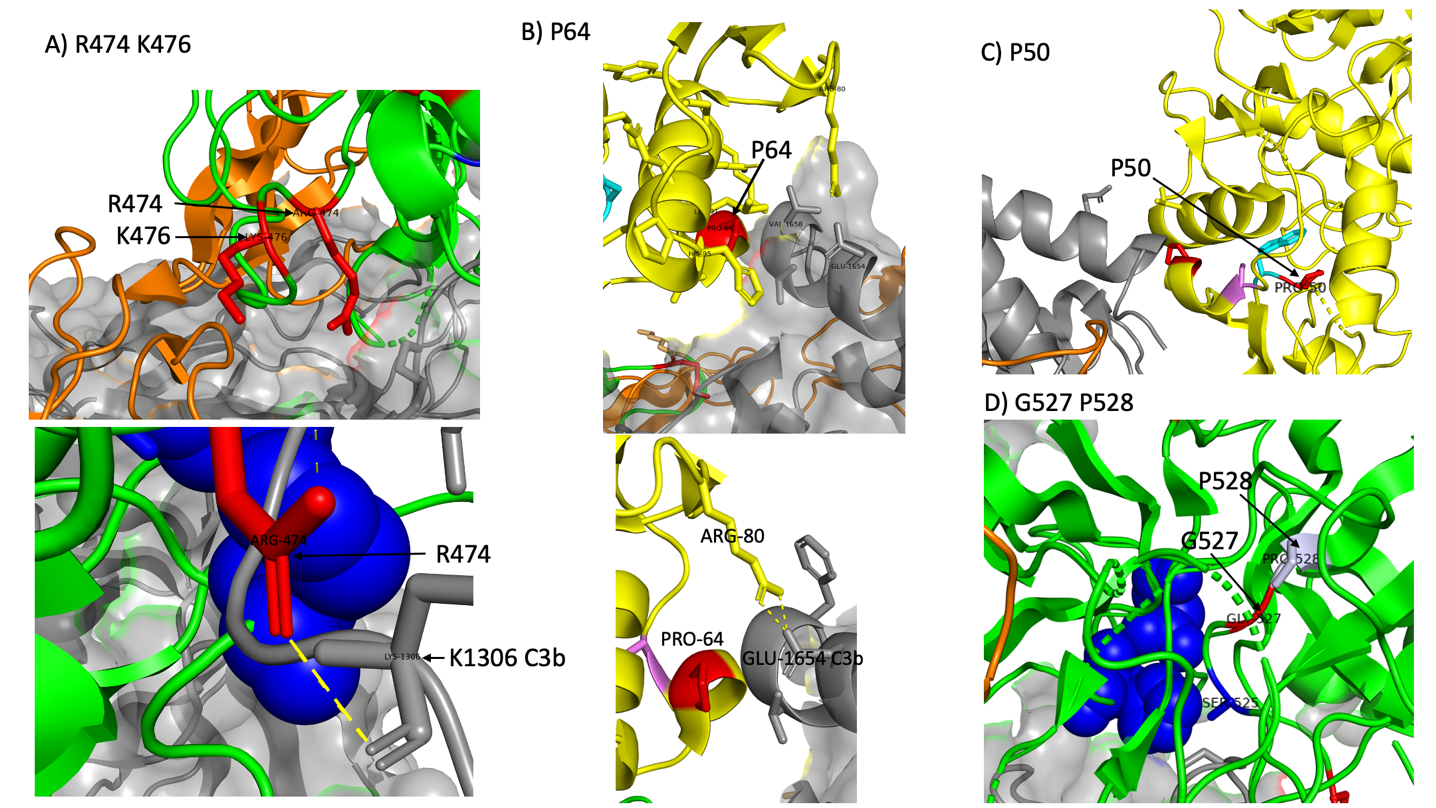
**

**Figure S6:** Three-dimensional modelling of Type II dysfunctional FI RVs. Type II dysfunctional FI RV side chains and their valence are shown by sticks in position within the 3D structure of the FI LC (green), HC (yellow) in complex with C3b (grey) and FH (orange) for A) P64; B) R474 and K476; C) P50A; D) G527 and P528. Polar interactions where present between molecules are shown by yellow dashes. This modelling was performed in and images were produced using PyMOL^TM^ v2.5.4 (Schrodinger, LLC) and PDB structure 5o32 (1).

**
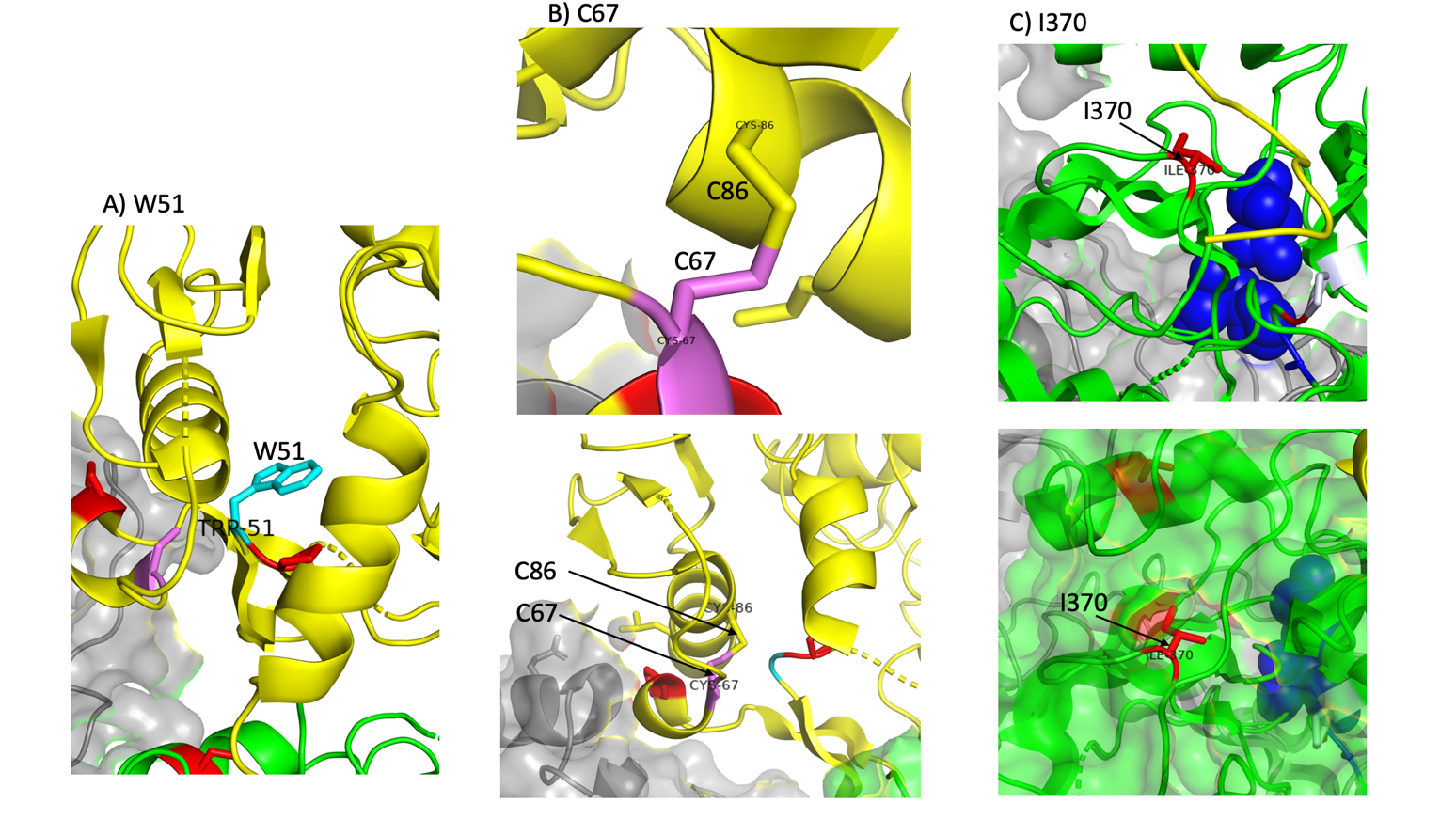
**

**Figure S7:** Three-dimensional modelling of Type I FI RVs. Type I FI RV side chains and their valence are shown by sticks in position within the 3D structure of the FI LC (green), HC (yellow) in complex with C3b (grey) and FH (orange) for A) W51; B) C67; C) I370. Polar interactions where present between molecules are shown by yellow dashes. This modelling was performed in and images were produced using PyMOL^TM^ v2.5.4 (Schrodinger, LLC) and PDB structure 5o32 (1).

| ***CFI* Rare Variant** | **CADD (PHRED)** | **REVEL score** |
| --- | --- | --- |
| p.Ser570Thr | 24.3 | 0.456 |
| p.Pro553Ser | 20.3 | 0.165 |
| p.Glu548Gln | 24.7 | 0.631 |
| p.Pro528Thr | 25.7 | 0.99 |
| p.Gly527Val | 26.4 | 0.958 |
| p.Ser525Ala | 25.5 | 0.853 |
| p.Lys476Glu | 24.1 | 0.38 |
| p.Arg474Gln | 24.2 | 0.55 |
| p.Lys442Gln | 15.45 | 0.304 |
| p.Lys441Arg | 6.753 | 0.505 |
| p.Ile370Thr | 25.2 | 0.963 |
| p.Arg339Gln | 23.1 | 0.699 |
| p.Thr203Ile | 12.48 | 0.04 |
| p.Cys67Arg | 26.6 | 0.925 |
| p.Pro64Leu | 26.3 | 0.703 |
| p.Trp51Ser | 25.9 | 0.603 |
| p.Pro50Ala | 23.5 | 0.654 |

**Table S1:** Comparison of combined annotation depletion dependent (CADD) score to REVEL score of variant deleteriousness. REVEL scores were acquired using dbNSFP v4 (2, 3) and a score of >0.5 indicates a ‘likely disease causing’ variant.

| **SCOPE subject** | | ***CFI* RV genotype** | **Serum FI level (µg/mL)** | **Amino acid** | **Literature Functional annotation (Type I, Type II, VUS, Benign)** | **Updated Functional annotation (Type I, Type II, VUS, Benign)** | **Final Annotation** |
| --- | --- | --- | --- | --- | --- | --- | --- |
| 1 | rs151142273, c65A>G, pTyr22Cys, heterozygous | | 29.1 | Tyr22Cys | VUS |  | VUS |
| 2 | no rsID, c134_135delAA, pLys45SerfsTer11, heterozygous | | 8.1 | Lys45SerfsTer11 | Type I |  | Type I |
| 3 | rs144082872, c148C>G, pPro50Ala, heterozygous | | Not available | Pro50Ala | VUS | Type II | Type II |
| 4 | rs144082872, c148C>G, pPro50Ala, heterozygous | | 20.037 | Pro50Ala | VUS | Type II | Type II |
| 5 | rs144082872, c148C>G, pPro50Ala, heterozygous | | Not available | Pro50Ala | VUS | Type II | Type II |
| 6 | rs144082872, c148C>G, pPro50Ala, heterozygous | | 19.8 | Pro50Ala | VUS | Type II | Type II |
| 7 | rs144082872, c148C>G, pPro50Ala, heterozygous | | 26.2 | Pro50Ala | VUS | Type II | Type II |
| 8 | rs144082872, c148C>G, pPro50Ala, heterozygous | | 17.6 | Pro50Ala | VUS | Type II | Type II |
| 9 | no rsID, c152G>C, pTrp51Ser, heterozygous | | 13.345 | Trp51Ser | VUS | Type I | Type I |
| 10 | rs773187287, c191C>T, pPro64Leu, heterozygous | | Not available | Pro64Leu | VUS | Type II | Type II |
| 11 | rs1727799010, c199T>C, pCys67Arg, homozygous | | Not available | Cys67Arg | VUS | Type I | Type I |
| 12 | rs200419722, c310G>A, pGly104Arg, heterozygous | | Not available | Gly104Arg | VUS |  | VUS |
| 13 | rs141853578, c355G>A, pGly119Arg, heterozygous | | 11.1 | Gly119Arg | Type I |  | Type I |
| 14 | rs141853578, c355G>A, pGly119Arg, heterozygous | | 10.5 | Gly119Arg | Type I |  | Type I |
| 15 | rs141853578, c355G>A, pGly119Arg, heterozygous | | 10.9 | Gly119Arg | Type I |  | Type I |
| 16 | rs141853578, c355G>A, pGly119Arg, heterozygous | | 8.1 | Gly119Arg | Type I |  | Type I |
| 17 | rs141853578, c355G>A, pGly119Arg, heterozygous | | 15.7 | Gly119Arg | Type I |  | Type I |
| 18 | rs141853578, c355G>A, pGly119Arg, heterozygous | | 9.7 | Gly119Arg | Type I |  | Type I |
| 19 | rs141853578, c355G>A, pGly119Arg, heterozygous | | 11.4 | Gly119Arg | Type I |  | Type I |
| 20 | rs141853578, c355G>A, pGly119Arg, heterozygous | | 12.8 | Gly119Arg | Type I |  | Type I |
| 21 | rs141853578, c355G>A, pGly119Arg, heterozygous | | Not available | Gly119Arg | Type I |  | Type I |
| 22 | rs141853578, c355G>A, pGly119Arg, heterozygous | | Not available | Gly119Arg | Type I |  | Type I |
| 23 | rs141853578, c355G>A, pGly119Arg, heterozygous | | 10.8 | Gly119Arg | Type I |  | Type I |
| 24 | rs141853578, c355G>A, pGly119Arg, heterozygous | | Not available | Gly119Arg | Type I |  | Type I |
| 25 | rs141853578, c355G>A, pGly119Arg, heterozygous | | Not available | Gly119Arg | Type I |  | Type I |
| 26 | rs141853578, c355G>A, pGly119Arg, heterozygous | | 9.6 | Gly119Arg | Type I |  | Type I |
| 27 | rs141853578, c355G>A, pGly119Arg, heterozygous | | Not available | Gly119Arg | Type I |  | Type I |
| 28 | rs141853578, c355G>A, pGly119Arg, heterozygous | | 1 | Gly119Arg | Type I |  | Type I |
| 29 | rs141853578, c355G>A, pGly119Arg, heterozygous | | 13.5 | Gly119Arg | Type I |  | Type I |
| 30 | rs141853578, c355G>A, pGly119Arg, heterozygous | | 12.3 | Gly119Arg | Type I |  | Type I |
| 31 | rs141853578, c355G>A, pGly119Arg, heterozygous | | Not available | Gly119Arg | Type I |  | Type I |
| 32 | rs141853578, c355G>A, pGly119Arg, heterozygous | | 10.8 | Gly119Arg | Type I |  | Type I |
| 33 | rs141853578, c355G>A, pGly119Arg, heterozygous | | 9.2 | Gly119Arg | Type I |  | Type I |
| 34 | rs141853578, c355G>A, pGly119Arg, heterozygous | | 7.6 | Gly119Arg | Type I |  | Type I |
| 35 | rs141853578, c355G>A, pGly119Arg, heterozygous | | 10.7 | Gly119Arg | Type I |  | Type I |
| 36 | rs141853578, c355G>A, pGly119Arg, heterozygous | | Not available | Gly119Arg | Type I |  | Type I |
| 37 | rs141853578, c355G>A, pGly119Arg, heterozygous | | 13.8 | Gly119Arg | Type I |  | Type I |
| 38 | rs141853578, c355G>A, pGly119Arg, heterozygous | | 9.1 | Gly119Arg | Type I |  | Type I |
| 39 | rs141853578, c355G>A, pGly119Arg, heterozygous | | Not available | Gly119Arg | Type I |  | Type I |
| 40 | rs141853578, c355G>A, pGly119Arg, heterozygous | | Not available | Gly119Arg | Type I |  | Type I |
| 41 | rs141853578, c355G>A, pGly119Arg, heterozygous | | Not available | Gly119Arg | Type I |  | Type I |
| 42 | rs141853578, c355G>A, pGly119Arg, heterozygous | | Not available | Gly119Arg | Type I |  | Type I |
| 43 | rs141853578, c355G>A, pGly119Arg, heterozygous | | Not available | Gly119Arg | Type I |  | Type I |
| 44 | rs141853578, c355G>A, pGly119Arg, heterozygous | | Not available | Gly119Arg | Type I |  | Type I |
| 45 | rs141853578, c355G>A, pGly119Arg, heterozygous | | Not available | Gly119Arg | Type I |  | Type I |
| 46 | rs141853578, c355G>A, pGly119Arg, heterozygous | | Not available | Gly119Arg | Type I |  | Type I |
| 47 | no rsID, c362C>A, pThr121Lys, heterozygous | | <3.6 | Thr121Lys | VUS | Type I | Type I |
| 48 | no rsID, c400C>G, pGln134Glu, heterozygous | | 37.5 | Gln134Glu | VUS |  | VUS |
| 49 | rs772044176, c452A>G, pAsn151Ser, heterozygous | | Not available | Asn151Ser | Type I |  | Type I |
| 50 | rs367677199, c454G>A, pVal152Met, heterozygous | | Not available | Val152Met | Type I | Type I | Type I |
| 51 | rs367677199, c454G>A, pVal152Met, heterozygous | | Not available | Val152Met | Type I | Type I | Type I |
| 52 | rs367677199, c454G>A, pVal152Met, heterozygous | | Not available | Val152Met | Type I | Type I | Type I |
| 53 | rs367677199, c454G>A, pVal152Met, heterozygous | | Not available | Val152Met | Type I | Type I | Type I |
| 54 | rs367677199, c454G>A, pVal152Met, heterozygous | | Not available | Val152Met | Type I | Type I | Type I |
| 55 | rs367677199, c454G>A, pVal152Met, heterozygous | | 15.9 | Val152Met | Type I | Type I | Type I |
| 56 | rs367677199, c454G>A, pVal152Met, heterozygous | | 9 | Val152Met | Type I | Type I | Type I |
| 57 | rs367677199, c454G>A, pVal152Met, heterozygous | | 1 | Val152Met | Type I | Type I | Type I |
| 58 | rs367677199, c454G>A, pVal152Met, heterozygous | | 13.3 | Val152Met | Type I | Type I | Type I |
| 59 | rs546607673, c485G>A, pGly162Asp, heterozygous | | 11.7 | Gly162Asp | Type I |  | Type I |
| 60 | rs753060374, c530A>T, pAsn177Ile, heterozygous | | Not available | Asn177Ile | Type I | Type I | Type I |
| 61 | rs143366614, c560G>A, pArg187Gln, heterozygous | | Not available | Arg187Gln | Benign |  | Benign |
| 62 | rs368615806, c559C>T, pArg187Ter, heterozygous | | 8.4 | Arg187Ter | Type I |  | Type I |
| 63 | no rsID, c563G>C, pGly188Ala, heterozygous | | 8.6 | Gly188Ala | Type I |  | Type I |
| 64 | rs138346388, c608C>T, pThr203Ile, heterozygous | | Not available | Thr203Ile | VUS | Benign | Benign |
| 65 | rs138346388, c608C>T, pThr203Ile, heterozygous | | 27.1 | Thr203Ile | VUS | Benign | Benign |
| 66 | no rsID, c674A>T, pAsp225Val, heterozygous | | 20.5 | Asp225Val | VUS |  | VUS |
| 67 | rs1726937797, c688G>A, pVal230Met, heterozygous | | 11 | Val230Met | Type I | Type I | Type I |
| 68 | rs146444258, c719C>G, pAla240Gly, heterozygous | | 11.4 | Ala240Gly | Type I |  | Type I |
| 69 | rs146444258, c719C>G, pAla240Gly, heterozygous | | 8.7 | Ala240Gly | Type I |  | Type I |
| 70 | rs146444258, c719C>G, pAla240Gly, heterozygous | | 9.5 | Ala240Gly | Type I |  | Type I |
| 71 | rs146444258, c719C>G, pAla240Gly, heterozygous | | Not available | Ala240Gly | Type I |  | Type I |
| 72 | rs146444258, c719C>G, pAla240Gly, heterozygous | | Not available | Ala240Gly | Type I |  | Type I |
| 73 | rs146444258, c719C>G, pAla240Gly, heterozygous | | Not available | Ala240Gly | Type I |  | Type I |
| 74 | rs146444258, c719C>G, pAla240Gly, heterozygous | | Not available | Ala240Gly | Type I |  | Type I |
| 75 | rs146444258, c719C>G, pAla240Gly, heterozygous | | Not available | Ala240Gly | Type I |  | Type I |
| 76 | rs146444258, c719C>G, pAla240Gly, heterozygous | | Not available | Ala240Gly | Type I |  | Type I |
| 77 | rs146444258, c719C>G, pAla240Gly, heterozygous | | Not available | Ala240Gly | Type I |  | Type I |
| 78 | rs146444258, c719C>G, pAla240Gly, heterozygous | | Not available | Ala240Gly | Type I |  | Type I |
| 79 | rs146444258, c719C>G, pAla240Gly, heterozygous | | Not available | Ala240Gly | Type I |  | Type I |
| 80 | rs146444258, c719C>G, pAla240Gly, heterozygous | | Not available | Ala240Gly | Type I |  | Type I |
| 81 | rs146444258, c719C>G, pAla240Gly, heterozygous | | Not available | Ala240Gly | Type I |  | Type I |
| 82 | rs146444258, c719C>G, pAla240Gly, heterozygous | | Not available | Ala240Gly | Type I |  | Type I |
| 83 | rs146444258, c719C>G, pAla240Gly, heterozygous | | Not available | Ala240Gly | Type I |  | Type I |
| 84 | rs121964916, c728G>T, pGly243Val, heterozygous | | 8.8 | Gly243Val | VUS | Type I | Type I |
| 85 | no rsID, c751A>G, pSer251Gly, heterozygous | | Not available | Ser251Gly | VUS |  | VUS |
| 86 | rs146444258, c719C>G, pAla240Gly, heterozygous | | 10.7 | Ala258Thr | Type I |  | Type I |
| 87 | rs146444258, c719C>G, pAla240Gly, heterozygous | | 9.6 | Ala258Thr | Type I |  | Type I |
| 88 | rs146444258, c719C>G, pAla240Gly, heterozygous | | 6.9 | Ala258Thr | Type I |  | Type I |
| 89 | rs146444258, c719C>G, pAla240Gly, heterozygous | | 8 | Ala258Thr | Type I |  | Type I |
| 90 | rs146444258, c719C>G, pAla240Gly, heterozygous | | Not available | Ala258Thr | Type I |  | Type I |
| 91 | rs146444258, c719C>G, pAla240Gly, heterozygous | | Not available | Ala258Thr | Type I |  | Type I |
| 92 | rs146444258, c719C>G, pAla240Gly, heterozygous | | Not available | Ala258Thr | Type I |  | Type I |
| 93 | rs112534524, c782G>A, pGly261Asp, heterozygous | | Not available | Gly261Asp | Benign |  | Benign* |
| 94 | rs112534524, c782G>A, pGly261Asp, heterozygous | | Not available | Gly261Asp | Benign |  | Benign* |
| 95 | rs112534524, c782G>A, pGly261Asp, heterozygous | | Not available | Gly261Asp | Benign |  | Benign* |
| 96 | rs112534524, c782G>A, pGly261Asp, heterozygous | | Not available | Gly261Asp | Benign |  | Benign* |
| 97 | rs112534524, c782G>A, pGly261Asp, heterozygous | | Not available | Gly261Asp | Benign |  | Benign* |
| 98 | rs112534524, c782G>A, pGly261Asp, heterozygous | | Not available | Gly261Asp | Benign |  | Benign* |
| 99 | rs112534524, c782G>A, pGly261Asp, heterozygous | | Not available | Gly261Asp | Benign |  | Benign* |
| 100 | rs112534524, c782G>A, pGly261Asp, heterozygous | | Not available | Gly261Asp | Benign |  | Benign* |
| 101 | rs112534524, c782G>A, pGly261Asp, heterozygous | | 9.7 | Gly261Asp | Benign |  | Benign |
| 102 | rs112534524, c782G>A, pGly261Asp, heterozygous | | 6.9 | Gly261Asp | Benign |  | Benign* |
| 103 | rs112534524, c782G>A, pGly261Asp, heterozygous | | Not available | Gly261Asp | Benign |  | Benign* |
| 104 | rs112534524, c782G>A, pGly261Asp, heterozygous | | Not available | Gly261Asp | Benign |  | Benign* |
| 105 | rs112534524, c782G>A, pGly261Asp, heterozygous | | Not available | Gly261Asp | Benign |  | Benign* |
| 106 | rs112534524, c782G>A, pGly261Asp, heterozygous | | Not available | Gly261Asp | Benign |  | Benign* |
| 107 | rs112534524, c782G>A, pGly261Asp, heterozygous | | 1 | Gly261Asp | Benign |  | Benign* |
| 108 | rs112534524, c782G>A, pGly261Asp, heterozygous | | 1 | Gly261Asp | Benign |  | Benign* |
| 109 | rs112534524, c782G>A, pGly261Asp, heterozygous | | 9 | Gly261Asp | Benign |  | Benign* |
| 110 | rs760688154, c786delA, pGly263AlafsTer37, heterozygous | | 9.3 | Gly263AlafsTer37 | Type I |  | Type I |
| 111 | rs760688154, c786delA, pGly263AlafsTer37, heterozygous | | 13.7 | Gly263fs | Type I |  | Type I |
| 112 | rs760688154, c786delA, pGly263AlafsTer37, heterozygous | | 12.2 | Gly263fs | Type I |  | Type I |
| 113 | rs200544168, c788G>T, pGly263Val, heterozygous | | Not available | Gly263Val | Benign |  | Benign* |
| 114 | rs200544168, c788G>T, pGly263Val, heterozygous | | Not available | Gly263Val | Benign |  | Benign* |
| 115 | no rsID, c853A>G, pIle285Val, heterozygous | | Not available | Ile285Val | VUS |  | VUS |
| 116 | rs182078921, c859G>A, pGly287Arg, heterozygous | | 11.4 | Gly287Arg | Type I |  | Type I |
| 117 | rs182078921, c859G>A, pGly287Arg, heterozygous | | 16.1 | Gly287Arg | Type I |  | Type I |
| 118 | rs182078921, c859G>A, pGly287Arg, heterozygous | | Not available | Gly287Arg | Type I |  | Type I |
| 119 | rs1381469349, c893delC, pSer298LeufsTer2, heterozygous | | Not available | Ser298LeufsTer2 | Type I |  | Type I |
| 120 | rs121964917, c949C>T, pArg317Trp, heterozygous | | Not available | Arg317Trp | Type II |  | Type II |
| 121 | no rsID, c980G>A, pCys327Tyr, heterozygous | | 12.6 | Cys327Tyr | Type I | Type I | Type I |
| 122 | rs773085612, c1016G>A, pArg339Gln, heterozygous | | 25.7 | Arg339Gln | Type II | Type II | Type II |
| 123 | rs773085612, c1016G>A, pArg339Gln, heterozygous | | 22.5 | Arg339Gln | Type II | Type II | Type II |
| 124 | rs773085612, c1016G>A, pArg339Gln, heterozygous | | 21.8 | Arg339Gln | Type II | Type II | Type II |
| 125 | rs762761680, c1015C>T, pArg339Ter, heterozygous | | 9.2 | Arg339Ter | Type I |  | Type I |
| 126 | rs762761680, c1015C>T, pArg339Ter, heterozygous | | Not available | Arg339Ter | Type I |  | Type I |
| 127 | rs769419740, c1019T>C, pIle340Thr, heterozygous | | Not available | Ile340Thr | Type II |  | Type II |
| 128 | rs769419740, c1019T>C, pIle340Thr, heterozygous | | Not available | Ile340Thr | Type II |  | Type II |
| 129 | rs769419740, c1019T>C, pIle340Thr, heterozygous | | Not available | Ile340Thr | Type II |  | Type II |
| 130 | rs769419740, c1019T>C, pIle340Thr, heterozygous | | Not available | Ile340Thr | Type II |  | Type II |
| 131 | rs769419740, c1019T>C, pIle340Thr, heterozygous | | 23.2 | Ile340Thr | Type II |  | Type II |
| 132 | rs781498531, c1066G>C, pAla356Pro, heterozygous | | 12.1 | Ala356Pro | Type I |  | Type I |
| 133 | rs781498531, c1066G>C, pAla356Pro, heterozygous | | 11.5 | Ala356Pro | Type I |  | Type I |
| 134 | rs200881135, c1071T>G, pIle357Met, heterozygous | | 12.4 | Ile357Met | Type I |  | Type I |
| 135 | rs200881135, c1071T>G, pIle357Met, heterozygous | | 13.4 | Ile357Met | Type I |  | Type I |
| 136 | rs200881135, c1071T>G, pIle357Met, heterozygous | | Not available | Ile357Met | Type I |  | Type I |
| 137 | rs200619905, c1085G>C, pGly362Ala, heterozygous | | 11.8 | Gly362Ala | VUS | Type I | Type I |
| 138 | rs1167888427, c1109T>C, pIle370Thr, heterozygous | | 11.09 | Ile370Thr | VUS | Type I | Type I |
| 139 | rs200447318,  c1187T>C, pVal396Ala, heterozygous | | 28.2 | Val396Ala | VUS |  | VUS |
| 140 | rs756096859, c1205C>T, pPro402Leu, heterozygous | | 21.5 | Pro402Leu | VUS |  | VUS |
| 141 | rs181729783, c1216C>T, pArg406Cys, heterozygous | | Not available | Arg406Cys | Benign |  | Benign |
| 142 | rs74817407, c1217G>A, pArg406His, heterozygous | | Not available | Arg406His | Benign |  | Benign |
| 143 | rs74817407, c1217G>A, pArg406His, heterozygous | | Not available | Arg406His | Benign |  | Benign |
| 144 | rs74817407, c1217G>A, pArg406His, heterozygous | | Not available | Arg406His | Benign |  | Benign |
| 145 | rs74817407, c1217G>A, pArg406His, heterozygous | | Not available | Arg406His | Benign |  | Benign |
| 146 | rs74817407, c1217G>A, pArg406His, heterozygous | | Not available | Arg406His | Benign |  | Benign |
| 147 | rs74817407, c1217G>A, pArg406His, heterozygous | | Not available | Arg406His | Benign |  | Benign |
| 148 | rs74817407, c1217G>A, pArg406His, heterozygous | | Not available | Arg406His | Benign |  | Benign |
| 149 | rs74817407, c1217G>A, pArg406His, heterozygous | | Not available | Arg406His | Benign |  | Benign |
| 150 | rs74817407, c1217G>A, pArg406His, heterozygous | | Not available | Arg406His | Benign |  | Benign |
| 151 | rs74817407, c1217G>A, pArg406His, heterozygous | | Not available | Arg406His | Benign |  | Benign |
| 152 | rs74817407, c1217G>A, pArg406His, heterozygous | | Not available | Arg406His | Benign |  | Benign |
| 153 | rs74817407, c1217G>A, pArg406His, heterozygous | | Not available | Arg406His | Benign |  | Benign |
| 154 | rs371432629, c1234G>A, pVal412Met, heterozygous | | 12.8 | Val412Met | Type I |  | Type I |
| 155 | rs371432629, c1234G>A, pVal412Met, heterozygous | | 11 | Val412Met | Type I |  | Type I |
| 156 | rs61733901, c1246A>C, pIle416Leu, homozygous | | Not available | Ile416Leu | VUS | Type I | Type I |
| 157 | rs61733901, c1246A>C, pIle416Leu, heterozygous | | 1 | Ile416Leu | VUS | Type I | Type I |
| 158 | rs121964912, c1253A>T, pHis418Leu, homozygous | | Not available | His418Leu | Type I |  | Type I |
| 159 | no rsID, c1258A>C pAsn420His, heterozygous | | 18 | Asn420His | VUS |  | VUS |
| 160 | rs780858107, c1283A>G pAsn428Ser, heterozygous | | Not available | Asn428Ser | VUS |  | VUS |
| 161 | rs1248287792, c1291G>A, pAla431Thr, heterozygous | | Not available | Ala431Thr | Type I |  | Type I |
| 162 | rs753578849, c1311dupA, pAsp438ArgfsTer8, heterozygous | | Not available | Asp438ArgfsTer8 | VUS |  | VUS |
| 163 | rs41278047, c1322A>G, pLys441Arg, heterozygous | | Not available | Lys441Arg | Benign | Benign | Benign |
| 164 | rs41278047, c1322A>G, pLys441Arg, heterozygous | | Not available | Lys441Arg | Benign | Benign | Benign |
| 165 | rs41278047, c1322A>G, pLys441Arg, heterozygous | | Not available | Lys441Arg | Benign | Benign | Benign |
| 166 | rs41278047, c1322A>G, pLys441Arg, heterozygous | | Not available | Lys441Arg | Benign | Benign | Benign |
| 167 | rs41278047, c1322A>G, pLys441Arg, heterozygous | | 18.9 | Lys441Arg | Benign | Benign | Benign |
| 168 | rs41278047, c1322A>G, pLys441Arg, heterozygous | | Not available | Lys441Arg | Benign | Benign | Benign |
| 169 | rs41278047, c1322A>G, pLys441Arg, heterozygous | | Not available | Lys441Arg | Benign | Benign | Benign |
| 170 | rs41278047, c1322A>G, pLys441Arg, heterozygous | | Not available | Lys441Arg | Benign | Benign | Benign |
| 171 | rs41278047, c1322A>G, pLys441Arg, heterozygous | | 24.4 | Lys441Arg | Benign | Benign | Benign |
| 172 | rs41278047, c1322A>G, pLys441Arg, heterozygous | | Not available | Lys441Arg | Benign | Benign | Benign |
| 173 | rs41278047, c1322A>G, pLys441Arg, heterozygous | | Not available | Lys441Arg | Benign | Benign | Benign |
| 174 | rs41278047, c1322A>G, pLys441Arg, heterozygous | | Not available | Lys441Arg | Benign | Benign | Benign |
| 175 | rs41278047, c1322A>G, pLys441Arg, heterozygous | | Not available | Lys441Arg | Benign | Benign | Benign |
| 176 | rs41278047, c1322A>G, pLys441Arg, heterozygous | | Not available | Lys441Arg | Benign | Benign | Benign |
| 177 | rs41278047, c1322A>G, pLys441Arg, heterozygous | | 24.6 | Lys441Arg | Benign | Benign | Benign |
| 178 | rs41278047, c1322A>G, pLys441Arg, heterozygous | | Not available | Lys441Arg | Benign | Benign | Benign |
| 179 | rs41278047, c1322A>G, pLys441Arg, heterozygous | | 17.7 | Lys441Arg | Benign | Benign | Benign |
| 180 | rs41278047, c1322A>G, pLys441Arg, heterozygous | | 25.4 | Lys441Arg | Benign | Benign | Benign |
| 181 | rs41278047, c1322A>G, pLys441Arg, heterozygous | | 16.2 | Lys441Arg | Benign | Benign | Benign |
| 182 | rs41278047, c1322A>G, pLys441Arg, heterozygous | | Not available | Lys441Arg | Benign | Benign | Benign |
| 183 | rs41278047, c1322A>G, pLys441Arg, heterozygous | | Not available | Lys441Arg | Benign | Benign | Benign |
| 184 | rs41278047, c1322A>G, pLys441Arg, heterozygous | | Not available | Lys441Arg | Benign | Benign | Benign |
| 185 | rs41278047, c1322A>G, pLys441Arg, heterozygous | | 16.2 | Lys441Arg | Benign | Benign | Benign |
| 186 | rs41278047, c1322A>G, pLys441Arg, heterozygous | | Not available | Lys441Arg | Benign | Benign | Benign |
| 187 | rs41278047, c1322A>G, pLys441Arg, heterozygous | | Not available | Lys441Arg | Benign | Benign | Benign |
| 188 | rs41278047, c1322A>G, pLys441Arg, heterozygous | | Not available | Lys441Arg | Benign | Benign | Benign |
| 189 | rs41278047, c1322A>G, pLys441Arg, heterozygous | | Not available | Lys441Arg | Benign | Benign | Benign |
| 190 | rs41278047, c1322A>G, pLys441Arg, heterozygous | | Not available | Lys441Arg | Benign | Benign | Benign |
| 191 | rs41278047, c1322A>G, pLys441Arg, heterozygous | | 28.8 | Lys441Arg | Benign | Benign | Benign |
| 192 | rs41278047, c1322A>G, pLys441Arg, heterozygous | | Not available | Lys441Arg | Benign | Benign | Benign |
| 193 | rs41278047, c1322A>G, pLys441Arg, heterozygous | | 29.5 | Lys441Arg | Benign | Benign | Benign |
| 194 | rs41278047, c1322A>G, pLys441Arg, heterozygous | | Not available | Lys441Arg | Benign | Benign | Benign |
| 195 | rs41278047, c1322A>G, pLys441Arg, heterozygous | | Not available | Lys441Arg | Benign | Benign | Benign |
| 196 | rs41278047, c1322A>G, pLys441Arg, heterozygous | | Not available | Lys441Arg | Benign | Benign | Benign |
| 197 | rs41278047, c1322A>G, pLys441Arg, heterozygous | | Not available | Lys441Arg | Benign | Benign | Benign |
| 198 | rs41278047, c1322A>G, pLys441Arg, heterozygous | | 21.3 | Lys441Arg | Benign | Benign | Benign |
| 199 | rs774830806, c1324A>G, pLys442Glu, heterozygous | | 26 | Lys442Glu | VUS |  | VUS |
| 200 | rs773442889, c1330T>G, pCys444Gly, heterozygous | | 11.4 | Cys444Gly | VUS |  | VUS |
| 201 | rs989210294, c1376A>C, pTyr459Ser, heterozygous | | Not available | Tyr459Ser | VUS |  | VUS |
| 202 | rs989210294, c1376A>C, pTyr459Ser, heterozygous | | 18 | Tyr459Ser | VUS |  | VUS |
| 203 | rs121964913, c1420C>T pArg474Ter, heterozygous | | 11.7 | Arg474Ter | Type I |  | Type I |
| 204 | rs121964913, c1420C>T pArg474Ter, heterozygous | | 9.4 | Arg474Ter | Type I |  | Type I |
| 205 | rs121964913, c1420C>T, pArg474Ter, heterozygous | | 11.2 | Arg474Ter | Type I |  | Type I |
| 206 | rs121964913, c1420C>T, pArg474Ter, heterozygous | | Not available | Arg474Ter | Type I |  | Type I |
| 207 | no rsID, c1426A>G pLys476Glu heterozygous | | 19 | Lys476Glu | VUS | Type II | Type II |
| 208 | rs754972981, c1429G>C, pAsp477His, heterozygous | | 7.6 | Asp477His | Type I | Type I | Type I |
| 209 | rs780759494, c1479C>A, pSer493Arg, heterozygous | | Not available | Ser493Arg | VUS |  | VUS |
| 210 | rs1436775364, c1580G>T, pGly527Val, heterozygous | | Not available | Gly527Val | VUS | Type II | Type II |
| 211 | no rsID, c1582C>A, pPro528Thr, heterozygous | | 16.7 | Pro528Thr | VUS | Type II | Type II |
| 212 | no rsID, c1593T>G pCys531Trp, heterozygous | | 5.5 | Cys531Trp | VUS | Type I | Type I |
| 213 | no rsID, c1625G>T, pGly542Val, heterozygous | | Not available | Gly542Val | VUS |  | VUS |
| 214 | rs7437875, c1642G>C pGlu548Gln, heterozygous | | 20.6 | Glu548Gln | VUS | Benign | Benign |
| 215 | no rsID, c1642delG, pGlu548LysfsTer26, heterozygous | | Not available | Glu548LysfsTer26 | VUS |  | VUS |
| 216 | rs113460688, c1657C>T, pPro553Ser, heterozygous | | Not available | Pro553Ser | Type II/VUS | Benign? | Benign** |
| 217 | rs113460688, c1657C>T, pPro553Ser, heterozygous | | Not available | Pro553Ser | Type II/VUS | Benign? | Benign** |
| 218 | rs113460688, c1657C>T, pPro553Ser, heterozygous | | Not available | Pro553Ser | Type II/VUS | Benign? | Benign** |
| 219 | rs113460688, c1657C>T, pPro553Ser, heterozygous | | 15.7 | Pro553Ser | Type II/VUS | Benign? | Benign** |
| 220 | rs113460688, c1657C>T, pPro553Ser, heterozygous | | Not available | Pro553Ser | Type II/VUS | Benign? | Benign** |
| 221 | rs113460688, c1657C>T, pPro553Ser, heterozygous | | Not available | Pro553Ser | Type II/VUS | Benign? | Benign** |
| 222 | rs113460688, c1657C>T, pPro553Ser, heterozygous | | Not available | Pro553Ser | Type II/VUS | Benign? | Benign** |
| 223 | rs113460688, c1657C>T, pPro553Ser, heterozygous | | 22.1 | Pro553Ser | Type II/VUS | Benign? | Benign** |
| 224 | rs113460688, c1657C>T, pPro553Ser, heterozygous | | Not available | Pro553Ser | Type II/VUS | Benign? | Benign** |
| 225 | rs113460688, c1657C>T, pPro553Ser, heterozygous | | Not available | Pro553Ser | Type II/VUS | Benign? | Benign** |
| 226 | rs113460688, c1657C>T, pPro553Ser, heterozygous | | 23.9 | Pro553Ser | Type II/VUS | Benign? | Benign** |
| 227 | rs113460688, c1657C>T, pPro553Ser, heterozygous | | Not available | Pro553Ser | Type II/VUS | Benign? | Benign** |
| 228 | rs113460688, c1657C>T, pPro553Ser, heterozygous | | Not available | Pro553Ser | Type II/VUS | Benign? | Benign** |
| 229 | rs754572081, c1661A>T, pGlu554Val, heterozygous | | Not available | Glu554Val | Type II |  | Type II |
| 230 | rs200973120, c1709G>C, pSer570Thr, heterozygous | | Not available | Ser570Thr | VUS | Benign | Benign |
| 231 | rs200973120, c1709G>C, pSer570Thr, heterozygous | | Not available | Ser570Thr | VUS | Benign | Benign |
| 232 | rs200973120, c1709G>C, pSer570Thr, heterozygous | | 30 | Ser570Thr | VUS | Benign | Benign |
| 233 | no rsID, c1714C>G, pHis572Asp, heterozygous | | Not available | His572Asp | VUS |  | VUS |
| 234 | rs146444258, c719C>G, pAla240Gly, heterozygous AND rs41278047, c1322A>G, pLys441Arg, heterozygous | | Not available | Ala240Gly / Lys441Arg | Type I / Benign | Type I / Benign | Type I / Benign |
| 235 | rs112534524, c782G>A, pGly261Asp, heterozygous AND rs768216926, c1636T>C pTrp546Arg, heterozygous | | Not available | Gly261Asp / Trp546Arg | Benign/VUS |  | Benign/VUS |
| 236 | rs778875276, c1307A>T, pLys436Ile, heterozygous AND rs765956155, c1421G>A, pArg474Gln heterozygous | | 9.7 | Lys436Ile / Arg474Gln | VUS/VUS | VUS/Type I/II | VUS/Type II |

**Table S2:** SCOPE cohort *CFI* genotypic characterization. All *CFI* RV genotypes (236 individuals) are described with associated serum FI level and updated catagorization of the CFI RV. *G261D is a known low false positive in FI serum level due to steric hinderance of antibody epitope (Khan et al. 2021 ((4))); ** P553S was benign on all assays apart from real-time C3b:FH:FI complex building assay in (Hallam et al. 2022 ((5)))

| **Variant** | **Forward Primer** | **Reverse Primer** |
| --- | --- | --- |
| P50A | ctcacctctcctgcgataaagtcttctgccag**g**catggcagagatgcattgagggcacc | ggtgccctcaatgcatctctgccatg**c**ctggcagaagactttatcgcaggagaggtgag |
| W51S | gccagccat**c**gcagagatgcattgagggcacctgtg | cacaggtgccctcaatgcatctctgc**g**atggctggc |
| P64L | gagggcacctgtgtttgtaaactac**t**gtatcagtgcccaaagaatgg | ccattctttgggcactgatac**a**gtagtttacaaacacaggtgccctc |
| C67R | gggcacctgtgtttgtaaactaccgtatcag**c**gcccaaagaatggcactgcagtg | cactgcagtgccattctttgggc**g**ctgatacggtagtttacaaacacaggtgccc |
| R339Q | gtggagttaaaaacagaatgcacattcgaaggaaac**a**aattgtgggaggaaagcg | cgctttcctcccacaatttg**t**ttccttcgaatgtgcattctgtttttaactccac |
| I370T | gtgggggaatttata**c**tggtggctgttgg | ccaacagccacca**g**tataaattcccccac |
| K442Q | gacggaaacaaa**c**aagattgtgagctgcctcgttccatccctgcctgtgtcccc | ggggacacaggcagggatggaacgaggcagctcacaatctt**g**tttgtttccgtc |
| R474Q | tggctggggac**a**agaaaaagataacg | cgttatctttttct**t**gtccccagcca |
| K476E | ggctggggacgagaa**g**aagataacgaaagagtcttttcacttcagtgggg | ccccactgaagtgaaaagactctttcgttatctt**c**ttctcgtccccagcc |
| S525A | cctgtaaaggggac**g**ctggaggccccttagtctgtatggatgc | gcatccatacagactaaggggcctccag**c**gtcccctttacagg |
| G527V | gcctgtaaaggggactctggag**t**ccccttagtctgtatggatgcc | ggcatccatacagactaagggg**a**ctccagagtcccctttacaggc |
| P528T | gcctgtaaaggggactctggaggc**a**ccttagtctgtatggatgcc | ggcatccatacagactaagg**t**gcctccagagtcccctttacaggc |
| S570T | ggccaattattttgactggatta**c**ctaccatgtagg | cctacatggtag**g**taatccagtcaaaataattggcc |

**Table S3:** Mutagenesis primers for point mutation generation within the *CFI* gene. HPLC-purified primers were purchased from Integrated DNA Technologies. Bold letters indicate mutated base pairs.

**Supplemental References:**

1. Xue, X., Wu, J., Ricklin, D., Forneris, F., Di Crescenzio, P., Schmidt, C. Q., Granneman, J., Sharp, T. H., Lambris, J. D., and Gros, P. (2017) Regulator-dependent mechanisms of C3b processing by factor I allow differentiation of immune responses. *Nature structural &amp; molecular biology*. **24**, 643–651

2. Liu, X., Li, C., Mou, C., Dong, Y., and Tu, Y. (2020) dbNSFP v4: a comprehensive database of transcript-specific functional predictions and annotations for human nonsynonymous and splice-site SNVs. *Genome Med*. 10.1186/s13073-020-00803-9

3. Liu, X., Jian, X., and Boerwinkle, E. (2011) dbNSFP: A lightweight database of human nonsynonymous SNPs and their functional predictions. *Hum Mutat*. **32**, 894–899

4. Khan, A. H., Sutton, J., Cree, A. J., Khandhadia, S., De Salvo, G., Tobin, J., Prakash, P., Arora, R., Amoaku, W., Charbel Issa, P., MacLaren, R. E., Bishop, P. N., Peto, T., Mohamed, Q., Steel, D. H., Sivaprasad, S., Bailey, C., Menon, G., Kavanagh, D., and Lotery, A. J. (2021) Prevalence and phenotype associations of complement factor I mutations in geographic atrophy. *Hum Mutat*. **42**, 1139–1152

5. Hallam, T. M., Cox, T. E., Smith-Jackson, K., Brocklebank, V., Baral, A. J., Tzoumas, N., Steel, D. H., Wong, E. K. S., Shuttleworth, V. G., Lotery, A. J., Harris, C. L., Marchbank, K. J., and Kavanagh, D. (2022) A novel method for real-time analysis of the complement C3b:FH:FI complex reveals dominant negative CFI variants in age-related macular degeneration. *Front Immunol*. 10.3389/fimmu.2022.1028760
